# A Martini Coarse Grained Model of Citrate-Capped Gold Nanoparticles Interacting with Lipid Bilayers

**DOI:** 10.1021/acs.jctc.1c00627

**Published:** 2021-09-07

**Authors:** Sebastian Salassi, Lucrezia Caselli, Jacopo Cardellini, Enrico Lavagna, Costanza Montis, Debora Berti, Giulia Rossi

**Affiliations:** † Department of Physics, University of Genoa, Via Dodecaneso 33, Genoa 16146, Italy; ‡ Department of Chemistry “Ugo Schiff”, University of Florence, Via della Lastruccia 3, Sesto Fiorentino, Florence 50019, Italy; § CSGI, Consorzio Sistemi a Grande Interfase and Department of Chemistry “Ugo Schiff” University of Florence, Via della Lastruccia 3, Sesto Fiorentino, Florence 50019, Italy

## Abstract

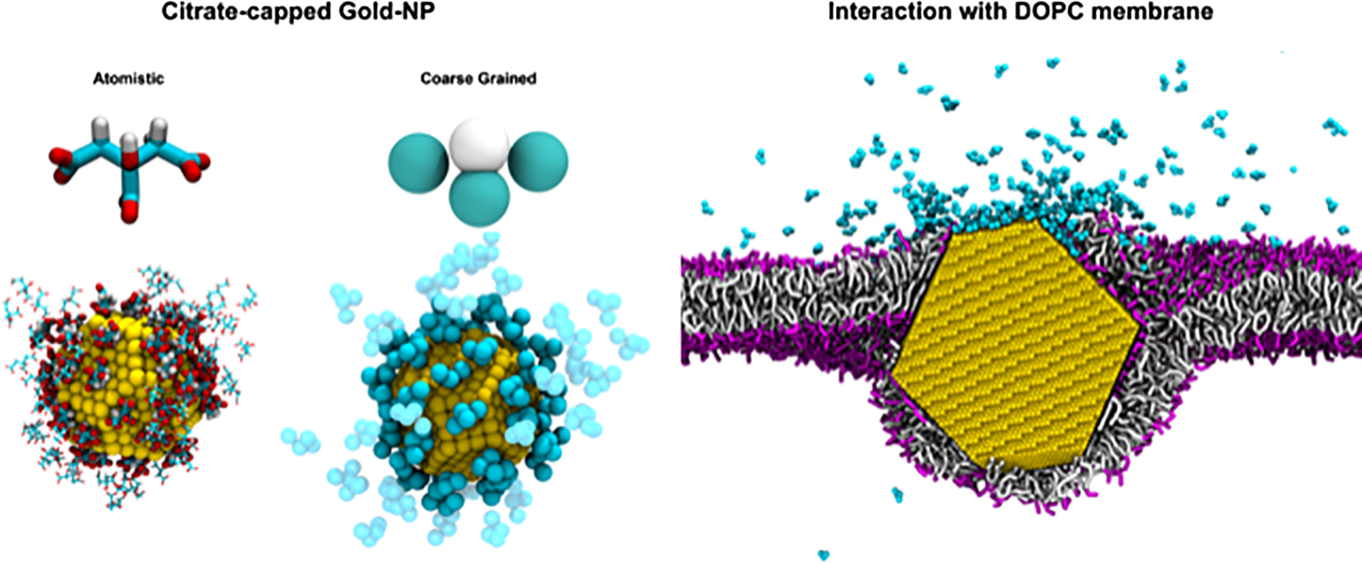

Citrate capping is one of the most common strategies to achieve the colloidal stability of Au nanoparticles (NPs) with diameters ranging from a few to hundreds of nanometers. Citrate-capped Au nanoparticles (CNPs) represent a step of the synthesis of Au NPs with specific functionalities, as CNPs can be further functionalized via ligand-exchange reactions, leading to the replacement of citrate with other organic ligands. In vitro, CNPs are also used to address the fundamental aspects of NP–membrane interactions, as they can directly interact with cells or model cell membranes. Their affinity for the bilayer is again mediated by the exchange of citrate with lipid molecules. Here, we propose a new computational model of CNPs compatible with the coarse grained Martini force field. The model, which we develop and validate through an extensive comparison with new all-atom molecular dynamics (MD) simulations and UV–vis and Fourier transform infrared spectroscopy data, is aimed at the MD simulation of the interaction between citrate-capped NPs and model phosphatidylcholine lipid membranes. As a test application we show that, during the interaction between a single CNP and a flat planar 1-palmitoyl-2-oleoyl-sn-glycero-3-phosphocholine bilayer, the citrate coating is spontaneously replaced by lipids on the surface of Au NPs, while the NP size and shape determine the final structural configuration of the NP–bilayer complex.

## Introduction

Nowadays, it is possible to functionalize Au NPs with a variety of organic ligands that confer them specific functionalities, such as the responsiveness to physical stimuli^1,2^ or the ability to selectively interact with specific biological targets.^3^ In the biomedical area, a strict requirement for Au NPs is to be dispersible and colloidally stable in aqueous environments. Indeed, colloidal stability is required from the synthesis stage to the final application. A common strategy to achieve colloidal stability is to cap the Au NP surface with sodium citrate, which can also be used as a reducing agent during nanoparticle (NP) synthesis.^4^ Citrate-capped Au NPs (CNPs) are commercialized with sizes ranging from a few to hundreds of nanometers in diameter. As citrate anions are noncovalently adsorbed on the Au NP surface, citrate-capped NPs can be used as a template for further surface functionalization: citrate molecules can be replaced with other ligands that bind more stably to the metal surface and provide the final desired functionality. Citrate can be replaced by aminoacids,^5^ thiols,^6−8^ lipids,^9,10^ amines, proteins, and nucleobases.^11^

Because of the wide spectrum of uses of CNPs, both for fundamental research and for biomedical applications, computer simulations have been used to elucidate the mode of binding of citrate to Au planar surfaces and NPs. Several efforts have been devoted to the development of citrate models with atomistic resolution, compatible with different versions of the CHARMM force field.^12−14^ The interaction between citrate, planar Au surfaces, and NPs has been addressed as well,^5,15,16^ also in combination with the GolP polarizable gold model.^17,18^ Within the sampling time accessible by atomistic simulations, these studies have described the possible adsorption configurations of monomeric citrate and citrate layers on Au surfaces and estimated their adsorption energies.^19^

The interaction between CNPs and the biological environment has been much less investigated at the molecular level. Experimental data suggest that CNPs can spontaneously and stably interact with model vesicles of phosphatidylcholines^20−23^ and that this interaction most likely implies the exchange of citrate molecules with lipids at the Au NP surface.^9,10,24^ Molecular dynamics (MD) simulations could provide important insights into the mechanism and thermodynamics of NP–membrane interaction. The size of NPs and vesicles in the experimental assays and the time scale of the evolution of NP–membrane complexes are difficult to match using models with atomistic resolutions, and a coarse grained (CG) approach would better fit the goal.

Very recently, Franco-Ulloa et al. have proposed an implicit ligand model of CNPs, aimed at describing the NPs’ colloidal stability as a function of pH and ionic strength.^25^ Here, we present the development of an explicit solvent and explicit ligand CG model of CNPs. Both the CG model of citrate and that of the Au NPs are parameterized to be compatible with the popular Martini force field.^26^ We performed model development, validation, and testing with the purpose of obtaining a reliable tool for the simulation of (i) citrate in aqueous solution, (ii) CNPs in polar and hydrophobic solvents, and (iii) CNPs in contact with phosphatidylcholine lipid membranes. We parameterized the citrate and Au CG models using as target properties structural and thermodynamic data obtained at all-atom resolution. We used UV–vis and Fourier transform infrared (FTIR) spectroscopy to assess experimentally the partitioning of CNPs in water–chloroform and water–chloroform– planar 1-palmitoyl-2-oleoyl-sn-glycero-3-phosphocholine (POPC) multiphase assays and used these experimental data to validate the model. Eventually, we tested the model by simulating the interactions of CNPs of various shapes (spherical and truncated octahedral) and sizes (2–14 nm) with POPC membranes. Our simulations show that lipids are readily exchanged at the surface of CNPs. Moreover, simulations shed light on the different molecular mechanisms that, depending on the NP size and shape, lead to full NP wrapping by fluid POPC bilayers.

## Methods

### Computational Methods

We performed the all-atom OPLS simulations (citrate in water, CNP in water and chloroform, Au planar surfaces, and CNPs on POPC lipid membranes) with the OPLS force field^27^ and the rigid SPC/E water model.^28^ More details of citrate, chloroform, and lipid atomistic models are reported in the Supporting Information (SI), together with their topologies. We remark here that we tested two alternative OPLS-compatible Au models. The first is the nonpolarizable gold model developed by Heinz and collaborators.^29^ The second is the polarizable gold model developed by Geada et al.,^30^ based on the previous model by Heinz.

#### Atomistic MD Setup

The cutoff of the van der Waals interaction was set to 1 nm, while the electrostatic interaction was treated via the long-range particle mesh Ewald method with a grid spacing of 0.12 nm. All bonds involving H atoms were constrained with the LINCS algorithm. All simulations were performed with a timestep of 2 fs, in the isothermal–isobaric (NpT) ensemble: temperature was kept constant at 300 K using a velocity rescale thermostat^31^ (τ_T_ = 1 ps); pressure was kept constant at 1 bar using a Berendsen barostat (τ_p_ = 1 ps) for the equilibration runs and using a Parrinello–Rahman barostat (τ_p_ = 1 ps) for the production runs. Compressibility was set at 4.5 × 10^–5^ bar^–1^. Most of the simulations were performed with isotropic pressure coupling. When the simulation box contained a planar lipid bilayer, we used a semi-isotropic pressure coupling to decouple in-plane and out-of-plane box deformations, unless otherwise stated.

#### CG MD Setup

The CG simulations were performed with the Martini force field.^32^ The cutoff of the van der Waals and electrostatic interactions was set to 1.1 nm and the dielectric constant to ε_r_ = 15. The time step was set to 20 fs. All simulations were performed in the NpT ensemble with temperature and pressure set to 300 K and 1 bar, respectively. The velocity rescale thermostat^31^ (τ_T_ = 1 ps) and Berendsen (τ_p_ = 4 ps) and Parrinello–Rahman (τ_p_ = 12 ps) barostats were used. Compressibility was set at 3 × 10^–4^ bar^–1^. Most of the simulations were performed in the isotropic pressure coupling. If a lipid bilayer was involved, we used the semi-isotropic pressure coupling, unless otherwise stated.

#### Enhanced Sampling Atomistic and CG Simulations

We used thermodynamic integration for the CG estimation of the water–octanol partitioning coefficient of citrate. We used umbrella sampling for the calculation of the potential of mean force (PMF) for the dimerization of the citrate in water (atomistic and CG) and for the calculation of the PMF of adsorbance of citrate on Au and on POPC (atomistic and CG). We used metadynamics to calculate the bidimensional free energy map of adsorption of POPC on Au (atomistic and CG). All the details concerning the setup of these biased simulations are described in Section 3 of the Supporting Information (Table S2 and related text).

#### Simulated Systems

The reader can find in the Supporting Information all the details of the simulated systems, such as composition of the simulation box, box sizes, initial configurations, and duration of the runs. Tables S1 and S2 list all the unbiased and biased simulations performed, respectively. All simulations were performed with Gromacs^33^ v. 2020.

#### Calculation of NP Coverage

The coverage of Au NPs by citrate molecules was computed by dividing the number of citrate molecules in direct contact with Au atoms with the area of the NP surface. The number of molecules in contact was computed with the ‘*mindist’* Gromacs tool by taking the citrate molecules that have the central carbon atom (the central bead in Martini) within a cutoff of 0.6 nm (0.8 nm in Martini) from an Au atom.

### Experimental Materials and Methods

#### Materials

Tetrachloroauric(III) acid (≥99.9%), trisodium citrate dihydrate (≥99.9%), 1-palmitoyl-2-oleoyl-sn-glycero-3-phosphocholine (POPC) (≥98.0%), and CHCl3 (≥99.9%) were provided by Sigma-Aldrich (St. Louis, MO). All chemicals were used as received. Milli-Q grade water was used in all preparations.

#### Synthesis of Au NPs

Anionic gold nanospheres of 15 nm in size were synthesized according to the Turkevich–Frens method. Briefly, 20 mL of a 1 mM HAuCl4 aqueous solution was brought to boiling temperature under constant and vigorous magnetic stirring. Two milliliters of 1% citric acid solution were then added, and the solution was further boiled for 20 min, until it acquired a deep red color. The NP dispersion was then slowly cooled down to room temperature.

#### Preparation of POPC Liposomes

An appropriate amount of lipid was dissolved in chloroform, and a lipid film was obtained by evaporating the solvent under a stream of nitrogen and overnight vacuum drying. The film was then swollen and suspended in warm (50 °C) Milli-Q water by vigorous vortex mixing, to a final 4 mg/mL lipid concentration. The resultant multilamellar vesicles in water were subjected to 10 freeze–thaw cycles and extruded 10 times through two stacked polycarbonate membranes with 100 nm pore size at room temperature, to obtain unilamellar vesicles with narrow and reproducible size distribution. Filtration was performed with the extruder (Lipex Biomembranes, Vancouver, Canada) through nucleopore membranes.

#### Water–Chloroform Biphasic Assay and Au NP Extraction by Centrifugation

An aqueous dispersion of CNPs was put in contact with an equal volume of chloroform containing POPC (at five different concentrations, 0, 0.01, 0.10, 0.50, and 1.0 mg/mL). The biphasic system was then either left in contact without any further action or centrifuged at 1500 rpm for 1 h with a high-speed Heraeus Biofuge stratos centrifuge equipped with a swing-out rotor, to facilitate the extraction of Au NPs to the CHCl_3_ phase. For noncentrifuged samples, the transfer of Au NPs to the CHCl_3_ phase was monitored through UV–vis, collecting the absorbance of the chloroform phase after 1 h. In case of centrifugation, the UV–vis spectrum of the chloroform phase was acquired before contact with Au NP dispersion in water, right after contact and after the centrifugation.

## Results and Discussion

### Development of a CG Model of Citrate

The atomistic model we chose as a target for the parameterization of the CG models is the all-atom OPLS force field.^27,34−36^ All the details concerning the OPLS citrate model development, its validation, and further comparisons between OPLS and alternative atomistic force fields are reported in the Supporting Information.

#### Mapping

**Figure 1:**
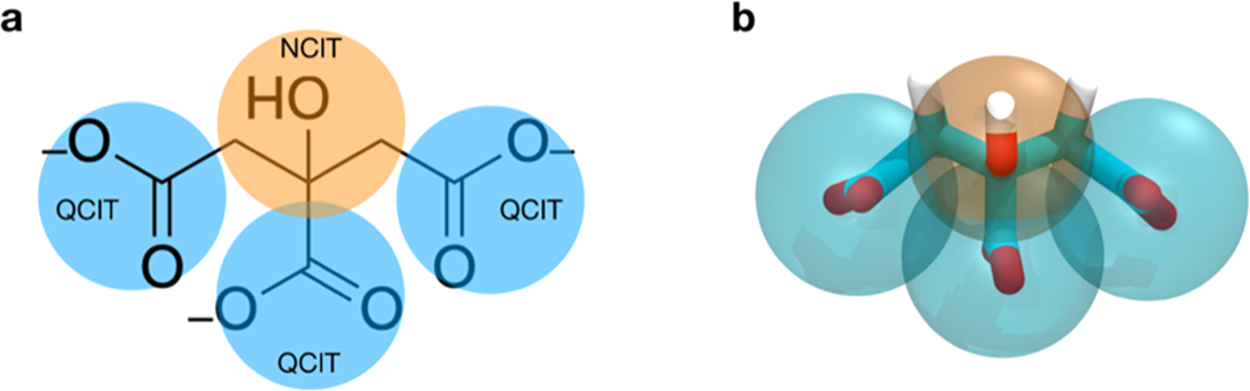
(a) Chemical structure of citrate with the superimposed Martini mapping. (b) Atomistic and Martini 3D view.

In Figure 1, the chemical structure of citrate and the proposed Martini mapping scheme are shown. CG citrate is modeled by four beads. We will name NCIT the bead representing the hydroxyl group, and QCIT the beads representing the carboxyl groups.

#### Citrate Bonded Interactions

The parameterization of the bonded interactions was based on the reproduction of the target atomistic distributions of distances, angles, and dihedrals, as shown in Figure S3 of the Supporting Information.

#### Citrate Nonbonded Interactions

As a first guess, we represented the hydroxyl group with an Nda Martini type, and the three carboxyl groups with a Qa Martini type, so as to capture the different charge state and polarity of the carboxyl and hydroxyl groups. We will refer to this first guess Martini model as the M3 model, as it bears a negative charge of −3*e*. We then tuned the nonbonded interactions (partial charges and Lennard-Jones (LJ) parameters) to reproduce three different target properties of citrate. We selected as target properties: *i.* the water–octanol partitioning coefficient of citrate, as suggested by the Martini parameterization approach;^32^*ii.* the citrate–citrate dimerization free energy profile obtained in water at all-atom resolution; and *iii.* the adsorption free energy profile of a single citrate molecule on a POPC model lipid bilayer. In the following, we describe the agreement between the predictions of the final model and these target properties. The set of final partial charges and LJ parameters of the new QCIT and NCIT beads, representing the carboxyl and hydroxyl groups, respectively, is listed in Table S4.

##### Water–Octanol Partitioning

The water–octanol partitioning coefficient of trisodium citrate is estimated as −1.3 and −0.55 by ALOGS^37^ and Chemicalize,^38^ respectively. At the end of the parameterization, the computed water–octanol partitioning coefficient^39,40^ of the CG citrate (with all charges set to zero^41^) is log(*P*^CG^) = −2, in satisfactory agreement with the estimated values.

##### Citrate–Citrate Interaction

**Figure 2:**
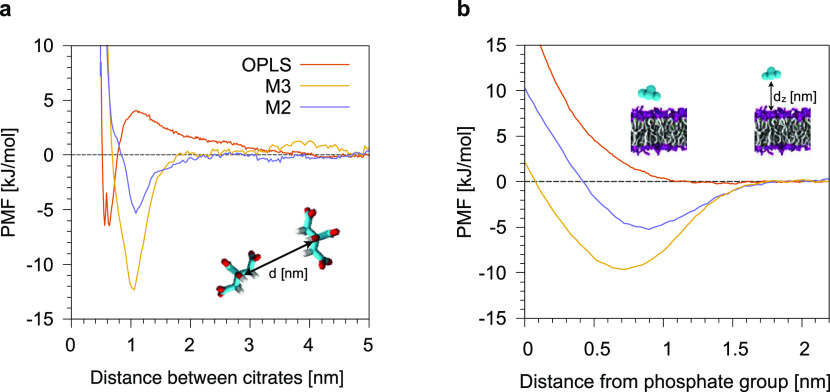
(a) PMF profiles of the dimerization of two citrate molecules in water. (b) PMF profiles of the adsorption of one citrate molecule on top of a zwitterionic model POPC lipid membrane. In red, the PMF profile obtained with the OPLS atomistic model, in violet, the one obtained with the Martini M2 model in which the citrate molecule is −2*e* charged, and in dark yellow, the Martini M3 model in which the citrate molecule is −3*e* charged. In the inset, lipid heads and tails are shown, respectively, as magenta and white sticks while citrate molecules in cyan.

To tune citrate–citrate interactions, we calculated the dimerization free energy profile, in water, at the atomistic level, and used it as a target for the development of the Martini model. The atomistic dimerization profile for the reference OPLS model is shown in Figure 2a. The free energy difference between the dimer and the unbound state is −7 ± 1 kJ mol^–1^ (∼ −3 *k*_B_*T*). When using the M3 model, in which citrate bears three negative electron charges, citrate molecules bind to each other too strongly. The main contribution to binding is due to counterion-mediated electrostatic interactions, as detailed in Supporting Information Figure S4. This is not surprising, as counterions in Martini are larger and stickier than their atomistic counterpart. We thus reduced the overall charge of the CG citrate to −2*e*, by attributing a charge of −0.7*e* to each QCIT bead and a charge of 0.1*e* to the NCIT bead. Concerning the LJ parameters for the interactions between QCIT, NCIT, water, and monovalent counterions, we treated QCIT and NCIT beads as Qa and Nda beads, respectively. We will refer to this model as the M2 model. We remark that, despite having a total charge of −2*e*, our CG model targets, here and in the rest of the model development, the behavior of a fully deprotonated citrate anion. The M2 model gives a better agreement with the atomistic data, with a free energy difference of Δ*G* = −5.3 ± 0.5 kJ mol^–1^. At the CG level, the dimer equilibrium distance is shifted by about +0.4 nm with respect to the atomistic model, as a result of the bulkiness of the Martini beads, whose LJ σ parameter is set to 0.47 nm irrespective of the chemical structure of the bead. Both CG models, as a consequence of the short-range treatment of Coulomb interaction, cannot capture the free energy barrier of about 4 kJ mol^–1^ (∼1.5 *k*_B_*T*) for dimerization that is present in the atomistic calculation. This discrepancy between the CG and atomistic model does not affect the equilibrium partitioning between the dimerized and dissolved states, but it implies a faster kinetic of binding and unbinding at the CG level.

##### Citrate–POPC Interaction

In Figure 2b, we show the adsorption free energy profile of a single citrate molecule on top of a neutral POPC membrane. The atomistic simulation does not show any stable bound state between the citrate molecule and the membrane. The same behavior is also shown for other small negatively charged and carboxylate-containing molecules, such as amino acids.^42^ The M3 model, on the contrary, predicts the existence of an adsorbed state. The free energy difference between the bound and unbound states is about −10 ± 2 kJ mol^–1^ (∼ −4 *k*_B_T) for the M3 model. This adsorbed state results from two contributions. The first is the electrostatic attraction between the negatively charged beads of citrate (bearing full −3*e* charge in M3) and the positively charged choline groups. We reduced this contribution using the M2 model in which citrate bears a −2*e* charge. The second contribution is due to LJ interactions. Our initial guess was to treat NCIT and QCIT beads as Nda and Qa beads, but we had to reduce NCIT-POPC and QCIT-POPC ε parameters to further reduce the depth of the CG PMF well. The citrate–citrate, citrate–water, and citrate–POPC interaction levels of the final M2 model are reported in Table S4. In the final model, the free energy difference between the adsorbed and dissolved states is −5 ± 2 kJ mol^–1^ (∼ −2 *k*_B_T) for M2, allowing for the dynamic attachment and detachment of citrate from the membrane surface at physiological temperature.

### Gold NP Model

The simulation of Martini solvents at the interface with solid surfaces calls for some caution. Because Martini solvents are LJ fluids, any nucleation site, such as an infinite flat surface or the finite facet of a NP, can increase the freezing temperature of the solvent and induce its crystallization at room temperature.^43,44^ We thus parameterized Au NPs in such a way as to reduce this artifact at the interface between the NP and Martini solvents.

#### Mapping

Our mapping of Au NPs follows a 1 to 1 mapping scheme: each Au atom is mapped onto a new Martini bead, called AU bead. The choice of a 1 to 1 mapping creates a significant lattice mismatch between the surface and Martini water, contributing to reduce the water freezing artifact.

#### AU–AU Interactions

The LJ parameters for AU–AU interactions are set to the Heinz nonpolarizable potential^29^ (σ_AU–AU_ = 0.2629 nm, ε_AU–AU_ = 22.14487 kJ mol^–1^), ensuring that the structure of the NP remains stable without the necessity to freeze or constrain Au atoms, which often leads to MD instabilities. AU beads are neutral, as they are in the atomistic model. Indeed, the choice of neutral AU beads seems reasonable for the specific case of CNPs, as X-ray photoelectron spectroscopy experiments^45^ have shown that Au atoms are mainly in the zero oxidation state when interacting with citrate molecules. Also ^23^Na nuclear magnetic resonance and transmission electron microscopy experiments suggest that Na^+^ ions are present at the citrate-capped Au surface, without charge transfer.^45^

#### AU–Water Interactions

The choice of a 1 to 1 mapping for AU requires that AU interactions with water are scaled down compared to the standard Martini, to compensate for the large surface density of the beads. We set the strength of AU–water interactions to ε_AU–W_ = 1 kJ mol^–1^ which, in combination with the use of antifreeze water, does not cause water freezing around Au NPs. σ_AU–P4_ was set to 0.401 nm, corresponding to the average distance between a cluster of four water molecules and the Au surface.

#### AU–Chloroform Interaction

At the Martini level, each chloroform molecule is mapped^32^ onto one bead of type C4. For the σ_AU–C4_, we used the standard Martini value, σ_AU–C4_ = 0.47 nm. The ε_AU–C4_ was chosen to reproduce the (atomistic) chloroform structuring around an Au NP with a core diameter of ∼2.6 nm. ε_AU–C4_ was set to 1.5 kJ mol^–1^. In Figure S5a,b, we show the atomistic and CG radial density function of water and chloroform, respectively, at the interface with an Au NP surface.

#### AU–Citrate Interaction

To set Au–citrate interactions at the CG level, we characterized them at the atomistic level. Here, we report the results obtained with the target OPLS force field.

**Figure 3:**
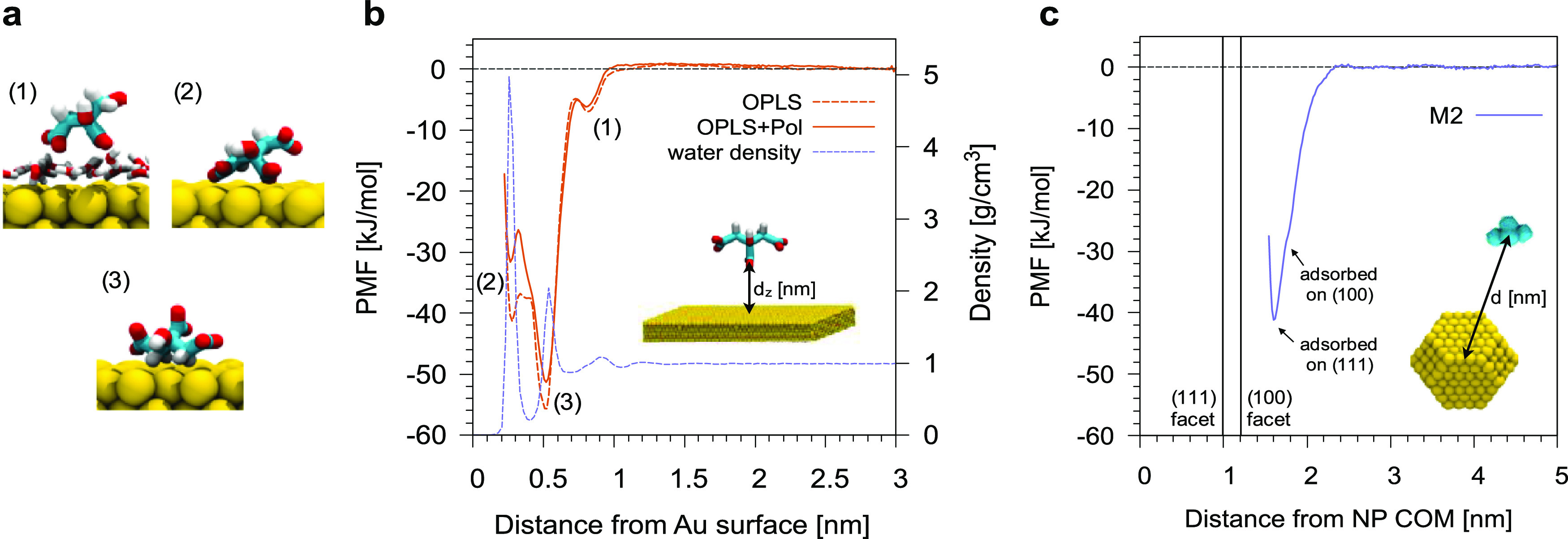
(a) Binding geometries of citrate on top of an Au (111) surface at the atomistic level. (b) PMF profile of the adsorption of one citrate molecule on an Au (111) surface with the OPLS force field. (c) PMF profile of the adsorption of one citrate molecule on an Au NP (diameter of ∼2.5 nm) with the Martini M2 model as a function of the distance from the NP COM. In both cases, the shaded area is the error on the PMF as calculated from the bootstrapping of trajectories.

First, we placed a single citrate molecule in water, above an Au (111) surface, and performed unbiased MD runs. Citrate spontaneously and stably bound to Au atoms with three possible binding geometries, as shown in Figure 3a. In configuration (1), a water layer is present between the gold surface and the oxygen atoms of the carboxylate groups. In configuration (2), the oxygen atoms of the carboxylate groups are in direct contact with the surface. Finally, in configuration (3), the CH_2_ groups of the citrate backbone lay on Au atoms.

The analysis of the unbiased simulations suggests that (1) and (2) binding geometries are metastable, while configuration (3) is the most stable. We calculated the adsorption free energy profile of a citrate molecule on an Au (111) surface, as shown in Figure 3b. All three binding geometries correspond to minima of the free energy profile. This result is robust with respect to the choice of the atomistic Au model: we obtained the same result with polarizable and nonpolarizable Au (Figure 3b). The free energy differences between the bound and unbound states are Δ*G*^1^ = −7 ± 1 kJ mol^–1^, Δ*G*^2^ = −41 ± 2 kJ mol^–1^, and Δ*G*^3^ = −56 ± 1 kJ mol^–1^ for the nonpolarizable OPLS force field, which we will use as a target for the refinement of citrate–gold CG interactions.

The adsorption of citrate on Au(111) surfaces has been investigated experimentally and by ab initio simulations, as well, suggesting that the most stable configurations for citrate on Au(111) are those in which one or more carboxylate oxygens are coordinated to Au,^45−48^ as in configuration (2). Configuration (2) is only metastable for OPLS (and totally absent for the C36 force field, as better detailed in Section 4 of the Supporting Information), a fact that may call for future refinements of the atomistic models. Here, as the difference between configurations (2) and (3) cannot be captured at the CG level, we did not investigate the matter further.

On the CG side, in Figure 3c we show the adsorption PMF of a citrate molecule on an Au NP. We tuned the nonbonded citrate-gold interactions for the M2 model until the agreement with the atomistic target was satisfactory; final parameters for AU–QCIT and AU–NCIT interactions are reported in Table S3.

#### AU–Lipid Interaction

**Figure 4:**
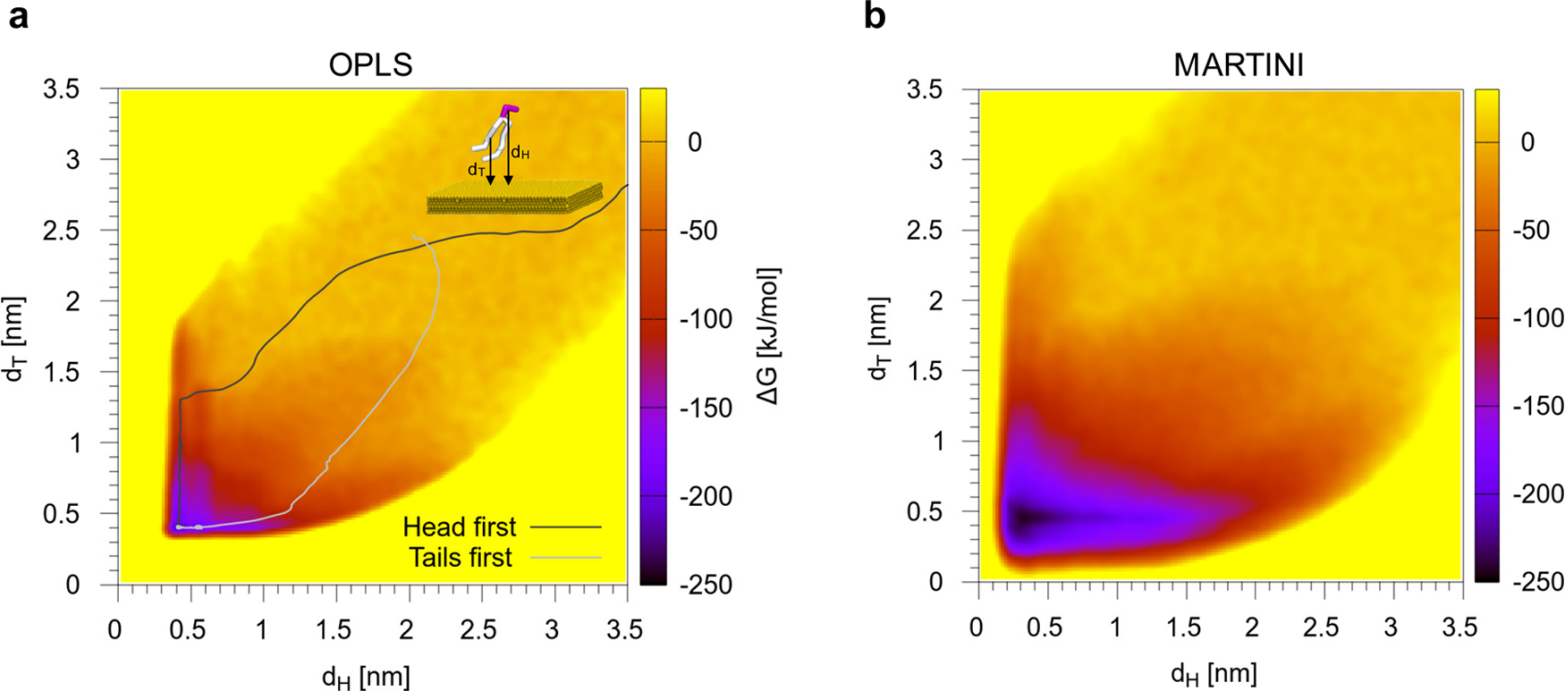
Adsorption free energy map of a single POPC lipid on Au for the OPLS force field (a) and the Martini CG model (b). *d*_H_ and *d*_T_ are the distances between the Au surface and the COM of the lipid head or tails, respectively.

We first studied the interaction between a POPC lipid and Au at the atomistic level and then used the result as a target for the parameterization of the CG AU–POPC interaction. We computed the adsorption free energy map of a single POPC lipid on an Au(111) surface, as shown in Figure 4a. The 2D free energy map was obtained by metadynamics using two collective variables: the distance between the gold surface and the COM of the lipid head (*d*_H_) and that between the gold surface and the COM of the lipid tails (*d*_T_).

POPC is readily adsorbed, and the configuration in which the lipid is lying flat on the gold surface corresponds to a deep free energy minimum of ∼220 kJ mol^–1^. Our optimized Au CG model provides a very similar free energy map, with an adsorption free energy of ∼245 kJ mol^–1^, which we consider in satisfactory agreement with the atomistic, given the overall strength of interaction.

It is interesting to look at the kinetics of adsorption, as POPC can follow different paths while approaching the Au surface. The leaf shape of the free energy map suggests a certain symmetry–both the lipid head and the tail can be adsorbed first. We performed 20 unbiased MD simulations, both at atomistic and CG levels, in which a POPC lipid spontaneously adsorbs on gold from the water phase. Each simulation had a different starting configuration. In Figure 4a, on top of the atomistic free energy map, two example paths are shown: one in which the first gold–lipid contact involves the lipid head, and another one in which the lipid tails are adsorbed in the first place. In Table 1, we sum up the results and show that for both the atomistic and the CG models the two mechanisms of adsorption are competitive. This observation is important to assess the hydrophilic versus hydrophobic nature of the gold surface. Moreover, it will have important repercussions on the mode of interaction between CNPs and zwitterionic lipid membranes, as we will see in the validation section.

**Table 1:** Adsorption Paths of POPC on Gold in Unbiased Atomistic and CG MD Runs

force field	no. of unbiased runs	head first	tail first	other
OPLS	21	11	7	3 (glycerol first, or head and tails together)
Martini	20	8	12	

### Model Validation

In this section, we validate the new models of citrate and gold NPs. We use different target properties and data obtained from atomistic simulations and experiments, as summed up in Table 2.

**Table 2:** Validation of Au and Citrate Models against Properties and Behavior of CNPs in Different Environments

interaction to be validated	target property/behavior	validation data
A. Au NP–citrate (in water)	adsorption and coverage	atomistic simulations (this work)
B. Au NP–POPC (in water)	POPC bilayer formation through different stages with increasing POPC coverage	scanning tunneling microscopy (STM) and atomic force microscopy (AFM) data;^49^
C. Au NP–POPC (in chloroform)	POPC monolayer formation on the Au NP surface	experimental data (this work)
D. CNP water/chloroform partitioning	Au NP partitions in water	experimental data (this work)
E. partitioning of CNPs in the water/POPC/chloroform mixture	CNPs migrate from water to chloroform by citrate release and POPC monolayer absorption	experimental data (this work)

#### Unbiased Adsorption of Citrate Molecules on Au NPs in Water

We performed a first set of validation runs looking at the unbiased adsorption of citrate on Au NPs.

**Figure 5:**
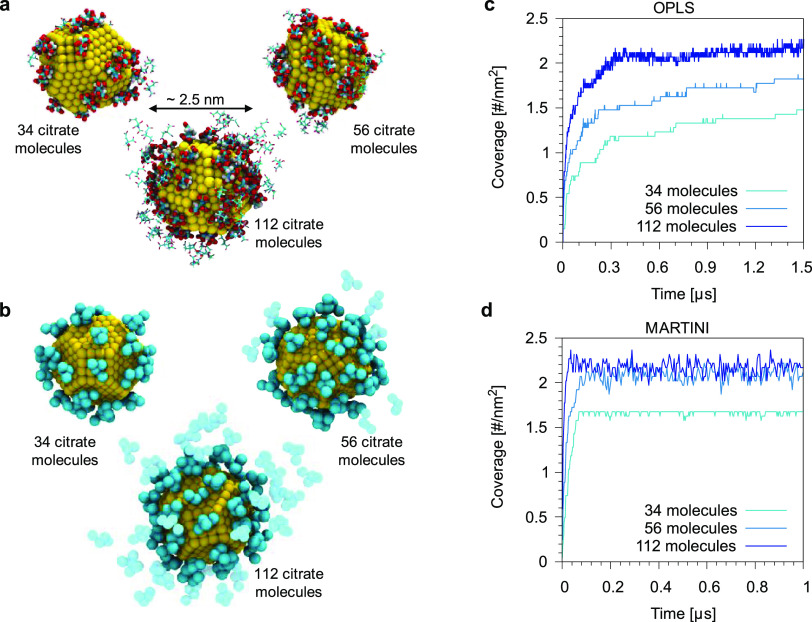
Final snapshots of the simulations of the spontaneous adsorption of citrate molecules on an Au NP (core diameter of ∼2.5 nm) with three different initial concentrations (34, 56, and 112 citrate molecules) for the atomistic OPLS (a) and for the CG Martini model (b). (c) and (d) Plot of the citrate coverage as a function of the simulation time for the different concentration. The coverage is expressed as the number of contacts between citrate and Au atoms, normalized by the surface area of the NP (∼20.3 nm^2^). Water is not shown.

In both atomistic and Martini simulations, we used a truncated octahedron Au NP with a core diameter of ∼2.5 nm, placed at the center of the simulation box and solvated with water. The citrate molecules (and their counterions) were randomly placed in the water phase. Atomistic and CG runs were built so as to have the same number of citrate molecules (34, 56, or 112) and the same overall citrate concentration (58, 95, and 187 mM, respectively). Figure 5 shows a snapshot of the final configuration at atomistic (panel a) and Martini levels (panel b) for the different concentrations. In both cases, citrate molecules favorably interact with the surface of the Au NP to form a stable complex.

At the atomistic level, we observe the formation of two shells of citrate molecules (see Figure 5 and the NP–citrate radial distribution functions in Figure S7). Even at the highest concentration, some portion of Au atoms are still exposed to water molecules. A similar behavior was also recently observed in the work of Perfilieva and collaborators.^15,17^ We remark that the Au–citrate contacts reported in Figure 5c are not yet fully converged after 1 μs, suggesting that a better coverage of the Au surface may be achieved on longer time scales. At the CG level, citrate adsorption is very similar. Irrespective of citrate concentration, the faster CG dynamics allows achieving the maximum coverage (Figure 5d) within the simulated timescale. Some citrate molecules remain in solution and can form a dynamic second layer of citrate molecules, as shown in Figure S7.

The equilibrium citrate coverage in the converged Martini simulations is between 1.6 and 2.3 molecules nm^–2^, depending on the overall citrate concentration. This coverage is in agreement with the available literature. Indeed, Park and Shumaker-Parry^47^ have estimated, via STM images of citrate-capped Au (111) surfaces, a coverage of ∼1.68 molecules nm^–2^, whereas Kunze et al.^50^ have determined via electrochemical analysis at pH = 3 a coverage of about 2.8 molecules nm^–2^. On the computational side, Phanchai and co-workers^51^ have estimated, using the atomistic Amber force field, a citrate coverage of ∼1.58 molecules nm^–2^ on a modified truncated octahedron Au NP with a core diameter of ∼2 nm.

#### POPC on Au, in Water

**Figure 6:**
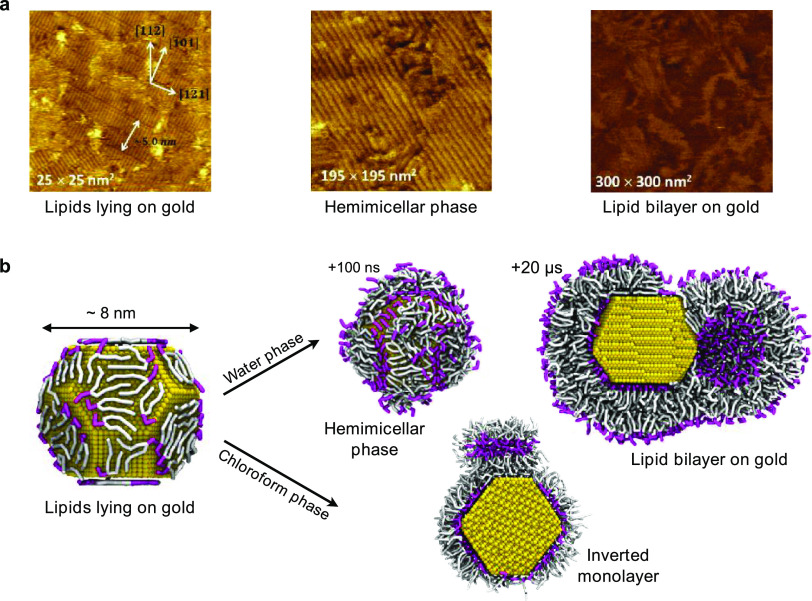
(a) EC-STM images of the different phases of the interaction between zwitterionic lipid vesicles (made of DMPC and cholesterol) and an Au (111) surface. Reprinted with permission from ref (49). Copyright 2015 American Chemical Society. (b) Snapshots from unbiased simulations of lipid adsorption and reorganization on the surface of an Au NP. Lipid heads and tails are shown, respectively, as magenta and white sticks, water or chloroform molecules are not shown for clarity.

Zwitterionic PC lipids readily adsorb on hydrophilic surfaces, such as mica or quartz. Unilamellar vesicles spread onto the hydrophilic surface, and their subsequent rupture leads to the formation of a supported lipid bilayer. In the presence of a gold substrate, the stabilization of a lipid bilayer is still possible as shown by Xu,^52^ but its mechanism of formation is different. Pawłowski and collaborators^49^ provided a detailed description of the lipid bilayer formation on Au by means of STM and AFM imaging and QCM measurements. The STM images of the different stages of bilayer formation are reported in Figure 6a. Initially (Figure 6a, left), the lipid vesicles interact with the Au surface and release lipids that adsorb on it. The lipids lie down on the Au surface and form ordered stripe-like domains in which lipids interact with each other in a head-tail-tail-head pattern. With the increase of lipid concentration, a hemimicellar film is formed (Figure 6a, center), which eventually facilitates the adsorption and rupture of further vesicles. Eventually (Figure 6a, right), a lipid bilayer is formed over the hemimicellar film, and the subsequent slow fusion of coupled layers leads to the formation of a single bilayer on the Au surface.

We used these data to validate our choice for the Au–POPC and Au–water interactions. We ran an unbiased simulation starting from a configuration in which a single 8 nm Au NP and 1500 POPC lipids are solvated in water (see Figure S8a). The system slowly transits between all intermediate states described by Pawłowski,^49^ as shown in Figure 6b, and the lipids finally form, after 20 μs, a full and stable lipid bilayer around the NP. The achievement of the equilibrium for the Au NP–POPC complex can be monitored by the convergence of the number of contacts between lipid heads and Au atoms, as shown in Figure S8c. We have estimated a lipid coverage of about 2.7 lipids nm^–2^ on the (111) facet and about 3.1 lipids nm^–2^ on the (100) facet. We remark that this validation of the model also offers an interpretation of the experimental data, suggesting that the competitive adsorption of lipid heads and tails on Au, in an aqueous environment, is a key physico-chemical driving force leading to the stabilization of these different metastable Au–POPC complexes.

#### Water–Chloroform CNP Partitioning: Experimental Results

**Figure 7:**
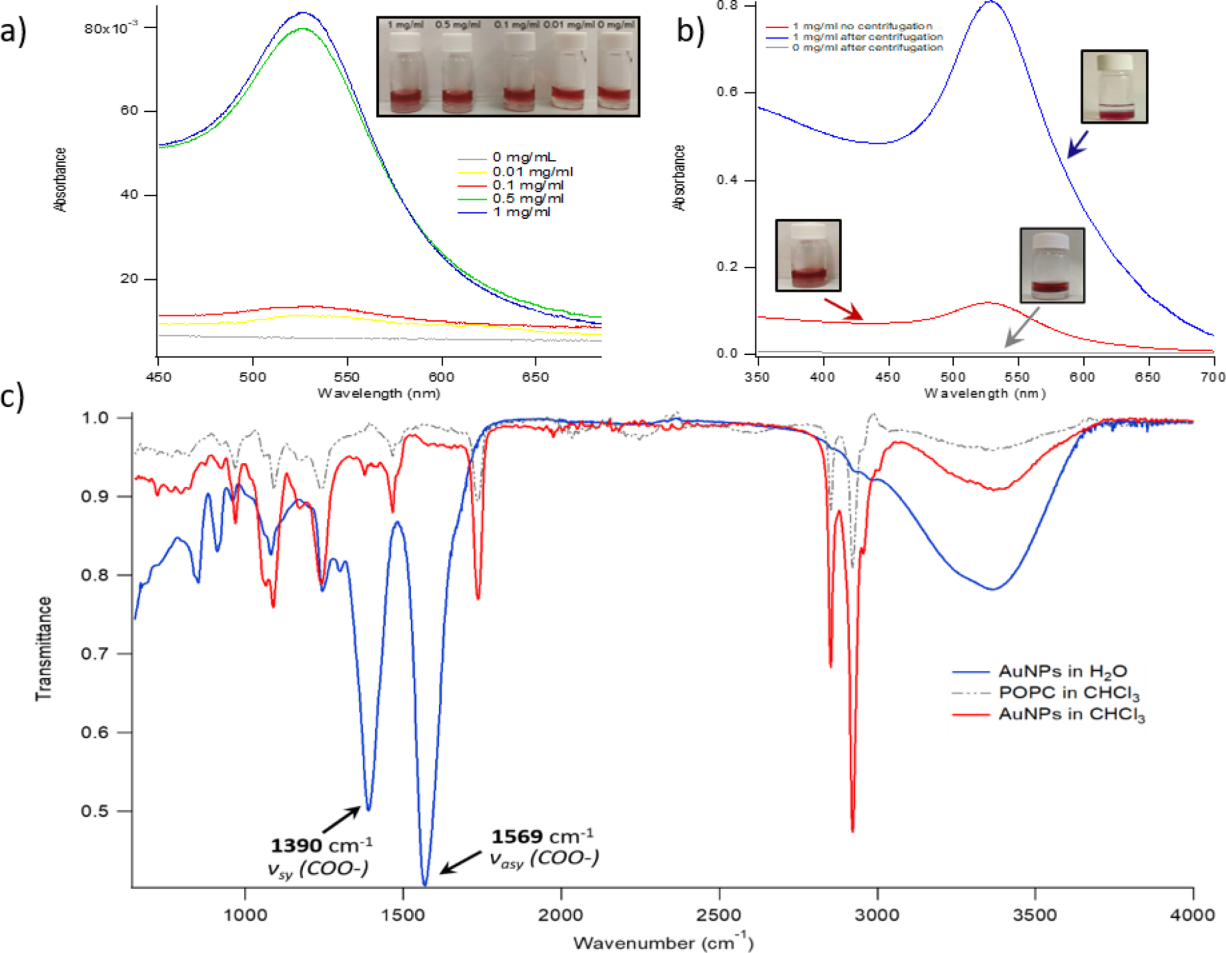
(a) UV–vis absorbance of the CHCl3 phase (after 1 h incubation with water-dispersed Au NPs) for different initial POPC concentrations (0, 0.01, 0.10, 0.50, and 1 mg/mL); inset: visual appearance of the corresponding samples; (b) UV–vis absorbance of the CHCl3 phase (1 mg/mL in POPC and 1 h contact with water-dispersed Au NPs) for centrifuged and noncentrifuged samples, and visual appearance of the corresponding samples; (c) ATR-FTIR spectra of CNPs recovered from a water dispersion, POPC, and AuNPs recovered from CHCl3 after extraction from the aqueous phase. The characteristic citrate features of the spectra of CNPs, that is, the νsy(COO−) and νasy(COO−) vibrations (arrowed), disappear when Au NPs are recovered from CHCl3 after extraction. The signals not originally present for CNPs are consistent with POPC (see the gray dashed spectrum of pure POPC for comparison).

To validate the interactions C., D., and E. reported in Table 2, we experimentally investigated the partition of CNPs between water and CHCl_3_ (either neat or containing POPC) in a biphasic 1:1 (v/v) system. In particular, 3 mL of a 4.3 nM aqueous dispersion of CNPs were put in contact with 3 mL of chloroform containing different amounts (0, 0.01, 0.10, 0.50, and 1.0 mg/mL) of POPC. Initially, the CHCl_3_ phase, with or without POPC, is transparent. At the same time, the aqueous dispersion is red thanks to the surface plasmon resonance (SPR) absorption of CNPs centered at 520 nm. After 1 h at room temperature without mechanical or magnetic stirring, the CHCl_3_ phase acquires a pale red color; the color intensity depends on the amount of POPC initially dissolved in the organic phase. In particular, in the absence of POPC, the organic phase remains transparent, while an increase of POPC concentrations from 0.01 to 1 mg/mL causes a gradual increase of the color intensity (see Figure 7a, inset), clearly consistent with a progressive migration of CNPs from the aqueous to the organic phase.

Figure 7a reports the UV–vis absorbance of the CHCl_3_ phase after contact with the CNP aqueous dispersion. In line with visual observations, no SPR signal is detected when POPC is not present in the organic phase (gray curve in Figure 7a). Conversely, if POPC is present, the spectrum of the organic phase exhibits the SPR absorption of CNPs. SPR intensity is clearly correlated with the lipid concentration in CHCl_3_, indicating that the presence of POPC promotes the transfer of CNPs to the organic phase.

Under these experimental conditions, that is, without mechanical or magnetic stirring, the phase transfer of CNPs is not complete after 1 h, and the water phase substantially preserves its original red color (Figure 7a, inset). However, the phase transfer is substantially fastened by mild centrifugation of samples right after contact (see the Methods section), Figure 7b. Interestingly, when the organic phase contains POPC, centrifugation induces a practically complete transfer of CNPs to CHCl_3_ over 1 h, as confirmed by the SPR increase with respect to the noncentrifuged sample and the resulting colorless appearance of the water phase (right inset in Figure 7b). Conversely, no significant differences are detected for the system without POPC before and after centrifugation. We interpret this result as because of the presence of a relatively small activation barrier to cross the interface, which can be overcome with an input of mechanical energy.

Because of the ligand’s anionic nature, CNPs are not dispersible in organic solvents, as confirmed in the reference extraction experiment with neat CHCl_3_; therefore, NPs’ transfer to CHCl_3_, promoted by the presence of POPC in the organic phase, must involve a reversal of the surface polarity of NPs, which results in an increased hydrophobic character. In a recent publication, a citrate–POPC ligand exchange, occurring at the CNP surface, was hypothesized to drive the observed modification in CNPs’ dispersibility. It is well known that the citrate anion is physisorbed on the NP surface through multiple weak interactions of nonspecific nature. Therefore, this ligand can be easily displaced at the water–CHCl_3_ interface, where a surface excess of POPC forms an oriented monolayer to minimize the CHCl_3_/water interfacial tension. The experimental data thus suggest that a POPC layer coats the NP surface, with the hydrophilic headgroup on the Au surface and hydrophobic tails pointing outward. This configuration would fully account for the NP affinity for CHCl_3_. The possible citrate displacement from the ligand shell of NPs in passing from the aqueous to the organic phase was investigated by means of attenuated total reflectance-Fourier transform infrared (ATR-FTIR) spectroscopy (Figure 7c).

Figure 7c displays the ATR-FTIR spectrum of CNPs recovered from the aqueous dispersion, with two prominent peaks at 1390 and 1569 cm^–1^ (blue spectrum); these signals are assigned to the symmetric and asymmetric stretching vibrations of the carboxylate group of the citrate, adsorbed on the CNP surface as the coating agent. The spectral fingerprint of citrate completely vanishes after extraction of the NPs to CHCl_3_ (red curve, 1 mg/mL POPC concentration). The new spectral features with respect to CNPs perfectly match the aliphatic C–H symmetric and asymmetric stretching vibrations of POPC (red dashed curve). These results clearly indicate the absence of citrate and its quantitative replacement by POPC at the NP surface.

#### POPC on Au, in Chloroform

The equilibrium complexes that form when lipids adsorb on gold depend on the hydrophilicity or hydrophobicity of the solvent. We prepared an Au NP solvated in chloroform and placed 500 POPC lipids at random positions in the simulation box (see Figure S8b). Lipids formed inverted micelles before/while adsorbing on the surface of the NP, but on a time scale of tens of microseconds (our run duration was 25 μs) they formed an almost perfect monolayer around the Au NP, as shown in Figure 6b. POPC coverage was the same as that obtained in water.

#### Water/Chloroform CNP Partitioning

We set up a two-phase water–chloroform simulation box and placed a CNP in water, as shown in Figure S9. We used a truncated-octahedron CNP with a core diameter of ∼8 nm and an initial citrate coverage of 1.4 molecule nm^–2^. Despite the low citrate coverage, which may favor an interaction of the Au surface with chloroform, after 5 μs we observe that the CNP remains in the water phase, with little if any interaction with the water/chloroform interface, in agreement with the experimental data.

#### CNP Partitioning in Water/POPC/Chloroform Mixtures

**Figure 8:**
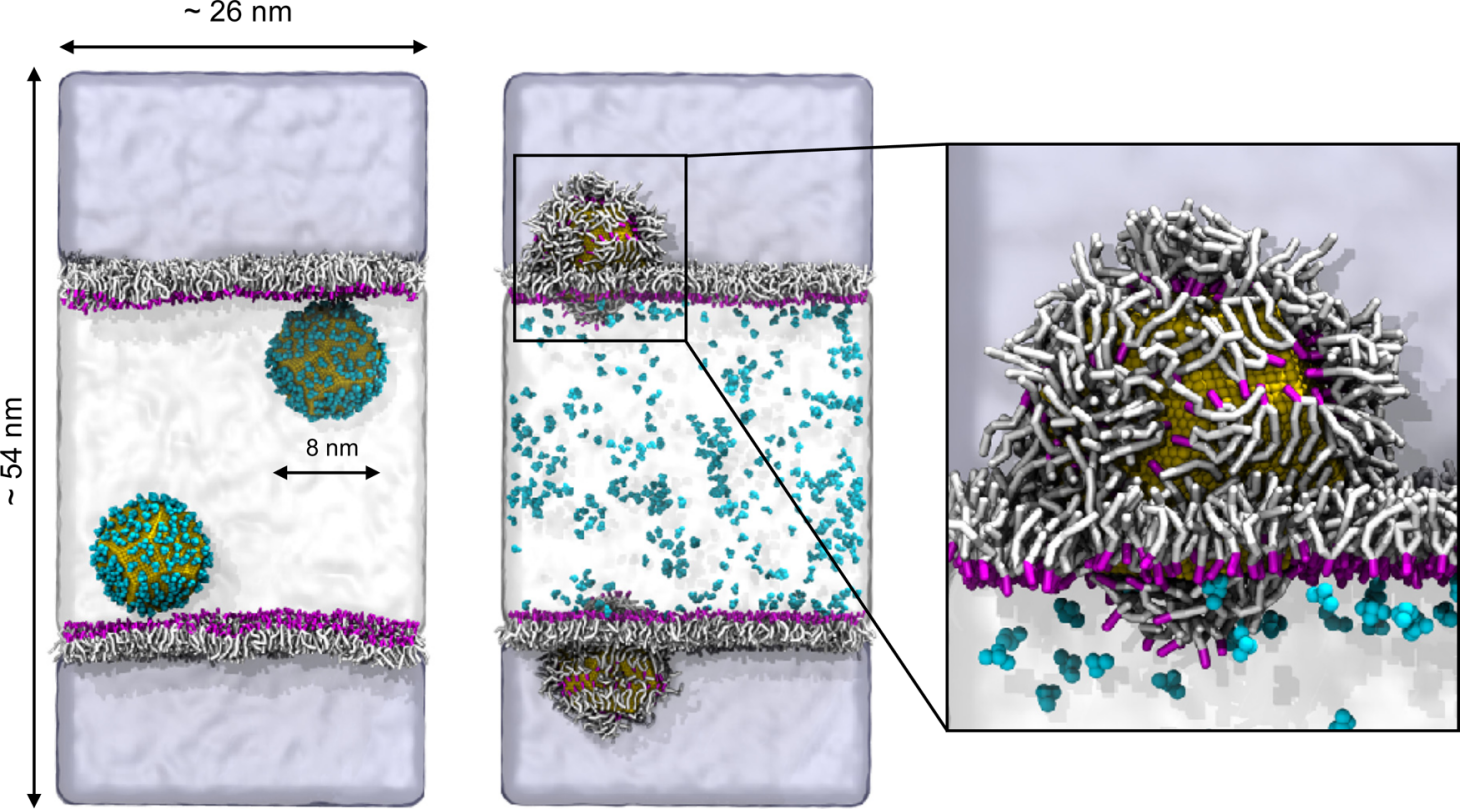
Water and chloroform are shown, respectively, as white and light-blue shaded areas. The citrate–lipid exchange reaction at the water/POPC/chloroform interface makes possible the spontaneous transfer of Au NPs from the water phase to the chloroform phase. In the right zoom panel, we can observe that in chloroform the lipid tails face the hydrophobic solvent, while the heads are in contact with the Au surface.

We then set up a system in which a POPC monolayer is present at the interface between water and chloroform and placed two CNPs in the water phase, as shown in Figure 8. As the unbiased MD simulation proceeded, we observed that the CNPs interacted with the POPC heads and they progressively exchanged citrate molecules, which were released in water, with lipid molecules. While the NPs were going deeper into the chloroform phase, their POPC coverage increased. After 5 μs, all the citrate molecules had been released in the water phase, and the NPs were completely covered by POPC lipids within the chloroform phase. We could not observe the complete detachment of the POPC-covered NP from the water/chloroform interface. As already suggested by the experimental tests, this process certainly requires the overcoming of large free energy barriers, and it is further inhibited in the simulation by the finite number of lipids in the simulation box. Again, the model reproduces reliably the partitioning of CNPs observed experimentally.

### Test Application: CNP–POPC Bilayer Interaction

We chose as a test application the interaction of a single CNP with a model POPC bilayer. At the atomistic level, the adsorption of CNPs on POPC is spontaneous (Figure S10 and related text), but the accessible time scales are limited to the observation of the very first CNP–membrane interaction stage only. At the CG level, we could study the CNP–membrane interaction with different NP types: we considered three truncated octahedron Au NPs with a core diameter of ∼2.5, ∼8, and ∼14 nm; and an Au NP with a spherical shape and a diameter of 11.2 nm.

**Figure 9:**
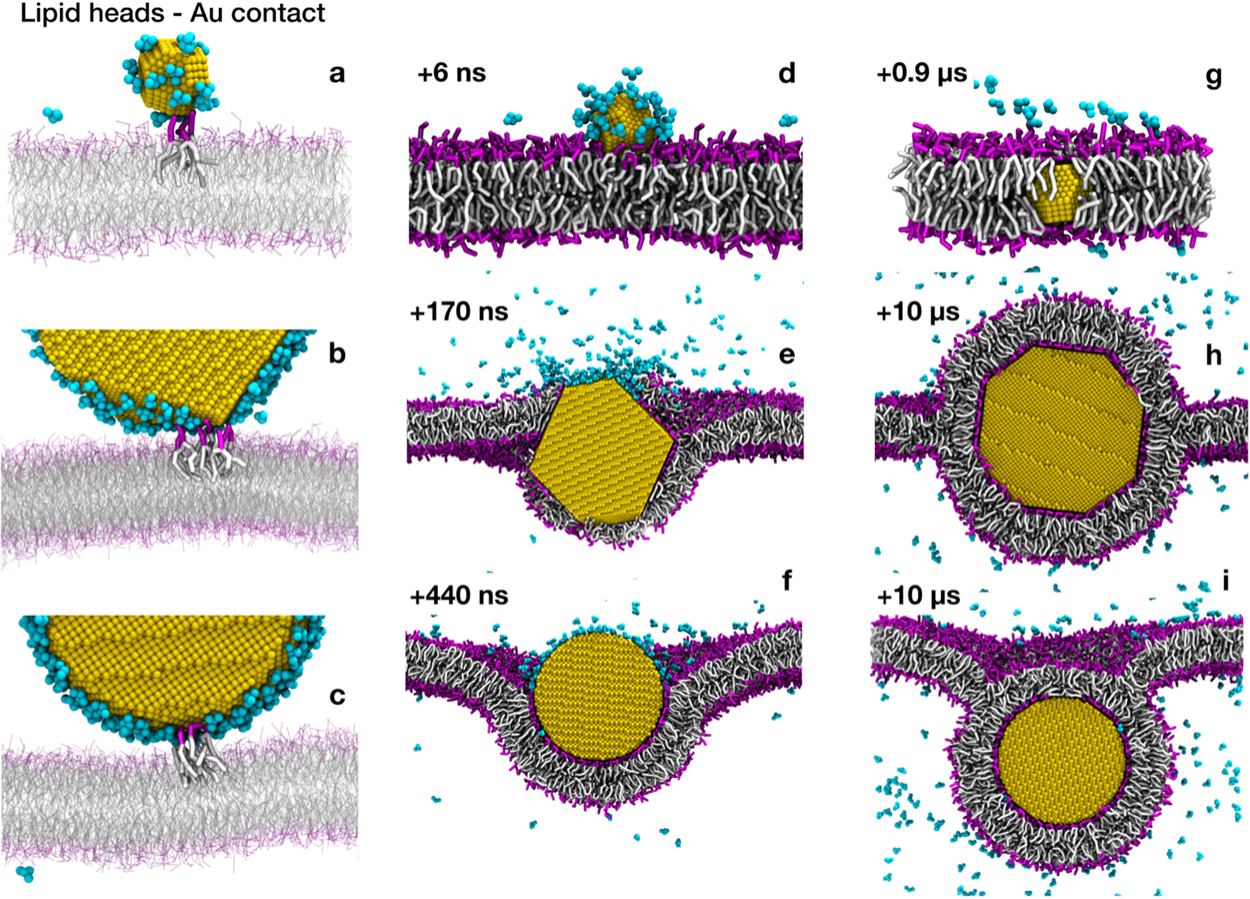
Time evolution of CNP–POPC bilayer interaction. Color code as in Figure 8. Top: 2.5 nm truncated octahedron CNP; middle: 14 nm truncated octahedron CNP; and bottom: 11.2 nm spherical CNP.

With all NP types, we observe that the CNP favorably interacts with the zwitterionic lipid bilayer. In the first stage of the interaction, the CNP stably adheres on top of the bilayer, while a layer of citrate molecules remains in between Au atoms and lipid head groups (see Figure S11a). The adsorbed state is metastable and its lifetime inversely correlates with the NP size and citrate coverage. As soon as the lipid head groups find their way to the Au surface, the citrate–lipid exchange reaction starts (see Figure 9a–c). Here, different mechanisms are observed depending on the NP size and shape. Small truncated octahedra pierce the hydrophobic core of the lipid membrane on a time scale of tens of nanoseconds (Figure 9d,g), without permanent alteration of membrane structure. For larger NPs, the interaction with the membrane is shape-dependent. Truncated octahedra (∼8 and ∼14 nm) are quite disruptive: the membrane core integrity is perturbed during the interaction with the NP. Both lipid heads and tails directly interact with the Au surface while citrate is released to the water phase (Figure 9e). Lipids then rearrange until the NP is completely covered by a lipid bilayer, a configuration in which only the lipid headgroups are adsorbed on the Au surface (Figure 9h). The time scale of this latter process ranged from a few to tens of microseconds, as shown in Figure S11b,c. At the end of the simulations, both the 8 and 14 nm octahedral NPs were completely covered and wrapped by the lipid bilayer. Spherical NPs with a core diameter of 11.2 nm (Figure 9c,f,i) deform, but do not pierce the bilayer within the simulated time. The NP wrapping proceeds until almost all citrate molecules are released to the water phase and the NP is completely covered by the bilayer, again on a time scale of several microseconds.

## Conclusions

In this paper we developed, validated, and tested a model for CNPs compatible with the popular Martini CG model, with the final goal to be able to simulate, by MD, the interaction between CNPs and model phospatidylcholine bilayers. We used, as the target for the model parameterization, structural and thermodynamic data obtained with the all-atom OPLS force field. We validated the model showing that it can reproduce (i) the collective kinetics of adsorption of citrate on Au NPs, as obtained in our atomistic simulations; (ii) the modes of adsorption of POPC lipids on Au, as reported by STM and AFM data;^49^ and eventually (iii) the partitioning of CNPs in multiphase water/chloroform and water/POPC/chloroform samples, as assessed by our UV–vis and FTIR spectroscopy assays.

We applied our model to the study of the interaction between a single CNP and a flat POPC bilayer. Our simulations show that, for all NP sizes and shapes considered, citrate is favorably replaced by lipids on the surface of Au NPs. The NP–membrane complexes that spontaneously form within the simulated time scale (tens of microseconds) depend on the NP size. The smallest NPs (2.5 nm in diameter) pierce the membrane and penetrate its core, exposing the bare Au surface to the hydrophobic lipid tails. On the contrary, large NPs (8 and 14 nm in diameter) reach a configuration in which they are fully wrapped by a POPC bilayer.

The results of our test simulations are consistent with the available experimental data. The agreement concerns the citrate–lipid exchange, the consequent citrate release in the water phase, and the stabilization of a NP–membrane configuration in which the lipid headgroups are in direct contact with the Au NP surface. These results are preliminary to a more systematic study of CNP–membrane interactions. Recent experiments have shown that NP collective behavior at the vesicle surface, though, depends on the membrane phase (fluid or gel).^24^ A possible interpretation of these experimental data is that different degrees of NP wrapping would induce different degrees of membrane-mediated NP aggregation.^9,24^ Our CNP model will allow to look at the molecular details of these NP–membrane interactions.

## Supporting Information Available

Atomistic models; simulated systems; enhanced sampling calculations; CG model development; model validation; and test application (PDF)
