# Supplementary material for: A Martini Coarse Grained Model of Citrate-Capped Gold Nanoparticles Interacting with Lipid Bilayers
Source: J Chem Theory Comput. 2021 Sep 7;17(10):6597–609. doi: 10.1021/acs.jctc.1c00627 (PMC8515808; doi:10.1021/acs.jctc.1c00627)
Supplement: Supplementary file 1 — ct1c00627_si_001.pdf [file ct1c00627_si_001.pdf]

A Martini coarse grained model of citrate-capped gold nanoparticles for biomedical applications

S. Salassi[1], L. Caselli,[2,3] J. Cardellini[2,3], E. Lavagna[1], C. Montis[2,3], D. Berti[2,3], G. Rossi[1]

[1] Department of Physics, University of Genoa, Via Dodecaneso 33, 16146 Genoa, Italy
[2] Department of chemistry, University of Florence, Via della Lastruccia 3, Sesto Fiorentino, 50019 Florence, Italy
[3] Consorzio Sistemi a Grande Interfase, Department of Chemistry, University of Florence, Sesto Fiorentino, Italy

Supplementary information

1. Atomistic models

1.1 Citrate

The atomistic citrate model is based on the OPLS all-atom forcefield[1–4]. Following the work of Wright and collaborators[5], we have also developed a CHARMM36[6,7] compatible forcefield of the citrate molecule to be compared with the OPLS one.

The OPLS all-atom (AA) model for the trisodium citrate relies on the standard OPLS-AA rules[1–4] (Figure S1 a,b). The OCCO dihedral comes from the alpha-methoxy-lactic acid parametrized in the CHARMM36 (C36) forcefield[6,7], while the CCCC backbone dihedral is adjusted to well reproduce the Car-Parrinello simulations[5] and *ab-initio* DFT data[5,8]. The remaining bonded parameters in OPLS-AA come from ref [5]. Partial charges are derived from other similar compounds, such as acetate for the carboxyl groups, alcohols for the –OH group and alkanes for $-CH_2-$ groups. The partial charges have been adjusted to reach a sum of $-3e$. For what concerns the non-bonded 1-4 pair interactions only the first and last atom involved in a dihedral interaction are included with a scaling factor of 0.5 for both Van der Waals and Coulomb interaction. Moreover, as discussed in ref.[5], to avoid the formation of a stable hydrogen bond between the hydrogen of the hydroxyl group and the oxygens of the central carboxyl group, the non-bonded interaction between these atoms are excluded.

All the main simulations at atomistic level are produced with the OPLS forcefield but, in order to give a comparison between different atomistic forcefields, we have also updated the CHARMM27 citrate forcefield developed by Wright and collaborators[5] to be compatible with C36[6]. The bonded interactions missing in C36 comes from ref.[5]. The partial charges come from similar compound (acetate, alkanes and the *tert*-butyl alcohol) already parametrized in C36. Again, to avoid the formation of a stable hydrogen bond between the hydrogen of the hydroxyl group and the oxygens of the central carboxyl group, the non-bonded interaction between these atoms are scaled by a factor of 0.3 for both Van der Waals and electrostatic interaction, as done in ref.[5].

The CHARMM simulations are performed with the C36 forcefield[6] and the CHARMM TIP3P water model[9].

To validate the atomistic models, we used two targets. The first is the citrate conformation in water, and the second is the dimerization free energy profile of two citrate molecules in water.

Citrate conformations in water. Figure S1b shows the two most probable conformations of a citrate molecule in water. In the first conformation, the backbone is fully extended, while in the second it is partially folded. Eventually, there is a last possible conformation in which the backbone is fully folded, as observed in ref. [5], which we never observe, neither with OPLS nor with C36. OPLS and C36 offer a coherent picture in terms of geometry. Our results are also in agreement with those obtained by Wright and collaborators[5] with the CHARMM27 forcefield.

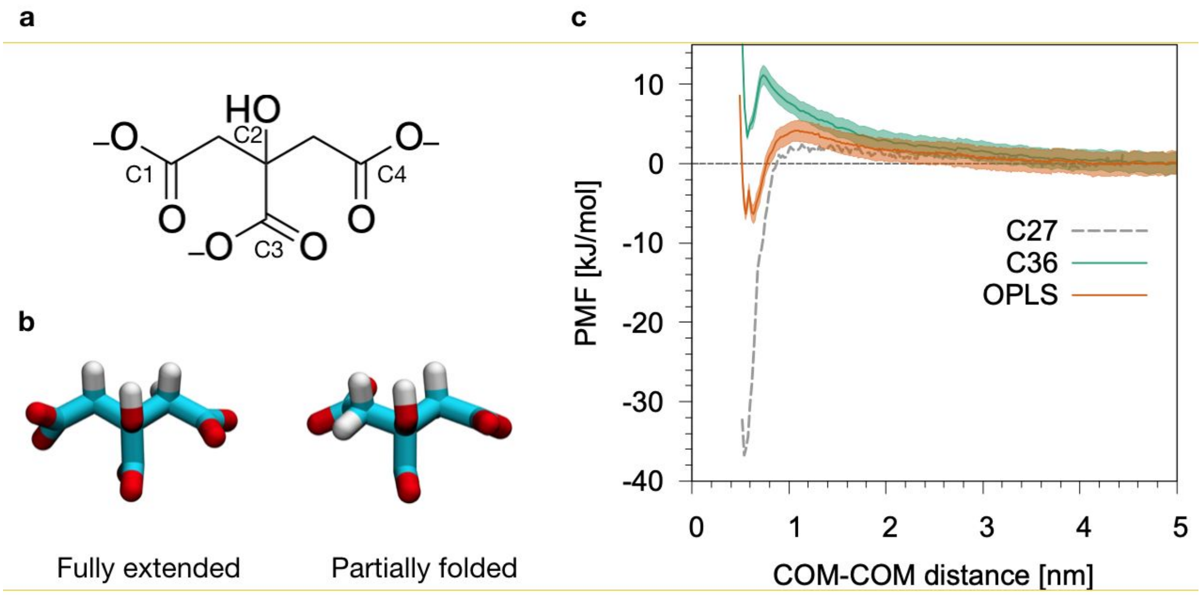

Figure S1. **a.** Chemical structure of the citrate molecule with the superimposed Martini mapping. **b.** The two possible conformations of a single citrate molecule in the water phase. Carbons in blue, oxygens in red and hydrogens in white. **c**. Dimerization potential of mean force (PMF) profile of two citrate molecules in the water phase obtained with the OPLS force field (orange), CHARMM27 (dashed gray) or CHARMM36 (green) forcefields. The shaded area is the error estimated with the bootstrapping of the trajectory. The PMF is obtained with the '*gmx wham*' tool.

Citrate dimerization free energy: comparison between OPLS and C36. It has been shown, with combined experimental and *ab-initio* data, that in citrate-capped gold NPs citrate molecules tend to form multiple layers around the NP: the first layer is composed of citrate molecules in direct contact with the Au surface while the external layers are formed by hydrogen bonding with the citrates of the more inner layers[10]. We calculated the dimerization free energy profile of two citrate molecules in water. As the profiles show (Figure S1c), with C36 the bound state is only metastable. On the contrary, the Wright model with CHARMM27 (gray-dashed curve in Figure S1c) predicts a strong binding, with a free energy difference of about –36 kJ mol^{-1}. The OPLS forcefield predicts an intermediate behavior, with a bound state that is slightly more stable than the unbound state ($\Delta G = -7 \pm 1$ kJ $mol^{-1} \sim -3\ k_B T$). Both models predict a free energy barrier between the bound and unbound states. In the OPLS case the barriers for dimerization and separation are of a few $k_B T$, allowing for the dynamic formation and disruption of small aggregates on simulated time scales. These different results can be explained considering the different amounts of Na^+ counterions that are stably bound

to the citrate molecule. In Figure S2 it is shown the radial distribution function (RDF) of Na^+ counterions with respect to citrate carboxyl oxygens. We observe that the C36 model has the lowest intensity peak, while the Wright model with CHARMM27 forcefield[6] has the highest one. We also measured the average number of contacts (within 0.6 nm) between citrate carboxyl oxygens and Na^+ counterions. The contacts are compatible to zero according to the C36 forcefield, meaning that on average less than 1 ion is bound to the molecule; according to OPLS, the contacts with ions are on average 1.5 ± 0.6, which can reduce the electrostatic repulsion during dimerization; lastly, the average number of ion-citrate contacts is 2.6 ± 0.5 for the CHARMM27 model. Thus with the CHARMM27 forcefield, almost all counterions are stably bound to the citrate molecules, explaining the strong propensity to aggregation that was observed in combination with the GoLP gold force field[11] in the work of Perfilieva and collaborators[12].

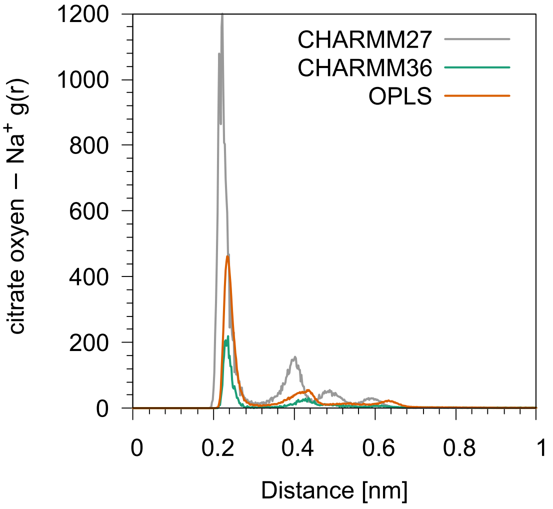

Figure S2. Radial distribution function *g(r)* of Na^+ counter ions around citrate molecules: comparison between the three forcefields.

We eventually opted for the OPLS model, which assures a stable citrate-citrate binding mode, in agreement with what observed in ref.[10], without overestimating the free energy of binding.

1.2 Chloroform

The chloroform OPLS model was based on the standard OPLS-AA rules[1–3] and it was generated with the *LibParGen* tool (http://zarbi.chem.yale.edu/ligpargen/) developed by the Jorgensen group[13–15] with partial charges set as the 1.14*CM1A-LBCC level of theory[13].

1.3 Lipids

For the POPC lipids we used the corrected united-atom Berger parameters[16,17].

1.4 Gold

Regarding the Au model at the atomistic level, we tested two forcefields. The first was developed by Heinz and collaborators[18]. In Heinz model Au atoms are Van der Waals interaction sites interacting through the Lennard-Jones (LJ) 12-6 potential. The LJ parameters were derived by a fit to the bulk (density and elastic moduli) and surface (solid/vapor surface tension and solid/water interface tension) properties of Au. The model does not take into account any polarization effect. An evolution of the Heinz model has been recently developed by Geada *et al.*[19], introducing Au polarizability at almost no computational cost. In this latter case, dummy electrons are coupled to Au atoms by a harmonic potential, while the LJ maintains the Heinz parameters.

The reader is referred to our online repository for the atomistic and CG topology and structure files[20] of citrate, chloroform, Au surfaces and NPs.

2. Simulated systems

2.1 Atomistic simulations

Citrate in water. Simulations of a single citrate molecule were performed in a box of about 4x4x4 nm^3 solvated with water. The simulation box for dimerization profiles was 11x11x11 nm^3 large and the system comprised 2 citrate molecules, their counterions and water.

Citrate on POPC. To study the adsorption profile of a citrate molecule on a model zwitterionic POPC lipid bilayer we equilibrated a lipid bilayer of 114 POPC lipids for 100 ns, and set up the simulation so that in the starting configuration the citrate molecule was placed above the membrane in the water phase.

Au surfaces and nanoparticles. We simulated both Au planar surfaces and nanoparticles. The non-polarizable Au (111) surface had an area of 3.4x3.4 nm^2 with 6 layers and a total of 720 Au atoms and it was obtained with the CHARMM-GUI *nanomaterial modeler* (http://www.charmm-gui.org)[21]. The polarizable Au (111) surface had an area of 4.7x4.9 nm^2 with 6 layers and a total of 1512 atoms; it was constructed repeating the unit cell provided by Geada *et al*[19]. The spherical NPs were obtained with a Python script using the MDAnalysis libraries[22,23] by taking all atoms within a certain distance from the NP COM. A python script, freely available in our repository, is used to generate a polarizable Au NP from a non-polarizable one.

Citrate on Au. The atomistic adsorption profile of a citrate molecule on Au was performed by placing a citrate molecule at non interacting distance from a Au (111) surface, and then solvating with water molecules. The atomistic adsorption profile of a POPC lipid on Au was obtained in the same way.

CNP on POPC bilayer. To study the interaction between a CNP and a model zwitterionic lipid bilayer at the atomistic level the box was composed by a preformed POPC lipid bilayer made of 456 lipids and a truncated octahedron Au NP with a core diameter of ~ 2.5 nm covered with 34 citrate molecules placed above the bilayer in the water phase. In all cases, the CNP was placed in the water phase above a pre-formed lipid bilayer. In all simulations counterions were present to neutralize the simulation box. All simulations were left equilibrating for at least 10 ns at the atomistic level.

2.2 CG simulations

Citrate in water. Simulations of a Martini single citrate molecule in water were run with a box of 6x6x6 nm^3.

Citrate on bilayer. As for other membrane simulations, the POPC bilayer was either preformed and already equilibrated or generated from scratch with the Insane[24] tool and then solvated and equilibrated for > 100 ns.

d_{NP} [nm]	# citrate molecules	# POPC lipids	Simulated time [μs]	Model
Unbiased adsorption of citrate molecules on Au NP				
2.5	34<br>56<br>112	–	1.5<br>1.5<br>1.5	OPLS + Au pol.

2.5	34<br>56<br>112	–	1<br>1<br>1	Martini
POPC self-assembly on the Au NP				
8	–	500	30	Martini (in chloroform)
8	–	1500	30	Martini (in water)
Biphasic system				
8	1.4 molecule/nm^2	–	5	Martini
8	1.4 molecule/nm^2	2x monolayer of 1024	5	Martini
Citrate-capped Au NP interacting with model lipid bilayer				
2.5	1.67 molecule/nm^2	456	2	OPLS + Au pol.
2.5	1.67 molecule/nm^2	456	2	OPLS
2.5	1.67molecule/nm^2	512	1	Martini
2.5	2.12 molecule/nm^2	512	1	Martini
8	1.36 molecule/nm^2	5408	15+10	Martini
11.2 spherical	1.26 molecule/nm^2	5408	10	Martini
14	1.37 molecule/nm^2	5408	10	Martini

Table S1. List of all unbiased simulations.

3. Enhanced sampling calculations

Thermodynamic integration. For the water-octanol partitioning free energy, we performed, following the method of Thermodynamic Integration (TI)[25] a series of 20 simulations, with λ values ranging from 0 to 1; the λ values were spaced at 0.05 or 0.1 depending on the steepness of the dG/d λ curve. Each simulation was equilibrated for 20 ns and run for 500 ns. The soft-core alpha parameter was set at 0.5, with the power and the radial power for lambda set respectively at 1 and 6, sigma at 0.3 and no coulombic soft-core transformation. The estimation of citrate water-octanol logP was done using the Bennett's acceptance ratio (BAR) method[26] with the '*gmx bar'* tool.

Umbrella Sampling. Most of the Potential of Mean Force (PMF) calculations were performed with the umbrella sampling[27] method and the PMF profiles were obtained with the *gmx wham* tool[28]. For the citrate-citrate dimerization profiles, the simulations were performed with an applied biasing force constant of 1250 kJ mol^{-1} nm^{-2} when using the atomistic models, and 1000 kJ mol^{-1} nm^{-2} when using Martini; the windows were spaced at 0.2 nm. For the citrate-POPC dimerization profile, we used a constant of 1250 kJ mol^{-1} nm^{-2} and a spacing of 0.15 nm with OPLS and 750 kJ mol^{-1} nm^{-2} and a spacing of 0.2 nm with Martini. For the Au_{111}-citrate adsorption PMF, the constant was set at 1250 kJ mol^{-1} nm^{-2} apart for the simulation with the CHARMM36 non polarizable model, where it was set at 1000 kJ mol^{-1} nm^{-2} and the windows were spaced at 0.15 nm; in the corresponding Martini dimerization profile, which was obtained with the 111 facets of an Au NP rather than with a flat surface, the force constant was 750 kJ mol^{-1} nm^{-2} and the window spacing was set at 0.2 nm. In certain cases, some windows were repeated with a higher pulling force, in order to obtain adequate sampling of high energy regions; in this case the range of the constant was 1250-12000 kJ mol^{-1} nm^{-2}. Pulling rates were always zero. Bootstrap analysis was performed with 80 bootstrap samples and tolerance set at $1 \cdot 10^{-6}$.

Metadynamics simulations. The bi-dimensional atomistic adsorption free energy map of a POPC lipid on top of a Au (111) surface was obtained with a well-tempered metadynamics[29,30] simulation. The Gaussians were deposited every 250 ps, their height was set to 6 kJ mol^{-1} with a variance of 0.04 nm, the temperature was set to 300 K and the bias factor to 60. The CG adsorption free energy map of a POPC lipid on a spherical Au NP with a core diameter of 8 nm was performed with a standard metadynamics simulation. The Gaussians were deposited every 500 ps, their height was set to 2.5 kJ mol^{-1} with a variance of 0.05 nm. The metadynamics runs were performed with Gromacs v. 2018 patched with Plumed v. 2.5[31]. A complete list of all biased simulations is summarized in Table S2 of the SI.

System	Forcefield	Total simulated time [µs]	Method
Citrate dimerization free energy profile	OPLS	0.5 + 2.6	US (26)
	CHARMM27	0.14 + 0.54	US (27)
	CHARMM36	0.5 + 2.6	US (26)
	Martini M3	1.3 + 7.8	US (25)
	**Martini M2**	**1.3 + 7.8**	**US (25)**
Citrate–POPC membrane free energy profile	OPLS	0.5 + 3.4	US (17)
	Martini M3	0.5 + 8.5	US (17)
	**Martini M2**	**0.5 + 8.5**	**US (17)**
Citrate–Au free energy profile	OPLS + Au Pol.	0.5 + 2.3	US (23)
	OPLS	0.4 + 2.2	US (22)
	CHARMM36	0.3 + 2.9	US (29)
	CHARMM36 + Au Pol.	0.2 + 2.3	US (23)
	**Martini M2**	**0.2 + 9.5**	**US (19)**
POPC–Au free energy map	OPLS + Pol.	5	Well-tempered metadynamics
	**Martini**	**15**	**Metadynamics**

Table S2. List of all biased simulations. The gray shaded cells are related to atomistic biased simulations used to parameterize the coarse-grained Martini models. The bolds rows are related to biased simulations performed with the final CG force field used in the validation stage. In the third column, the total simulated time (equilibration + production run) is shown. Numbers in parenthesis are the total number of windows used in umbrella sampling (US) simulations.

The starting configuration of each run can be given upon request. All simulations were performed with Gromacs[32] v. 2020.

4. CG model development

4.1 Development of the CG citrate model

Bonded distributions. Atomistic distributions were obtained from a trajectory of a single citrate molecule in water plus counter-ions.

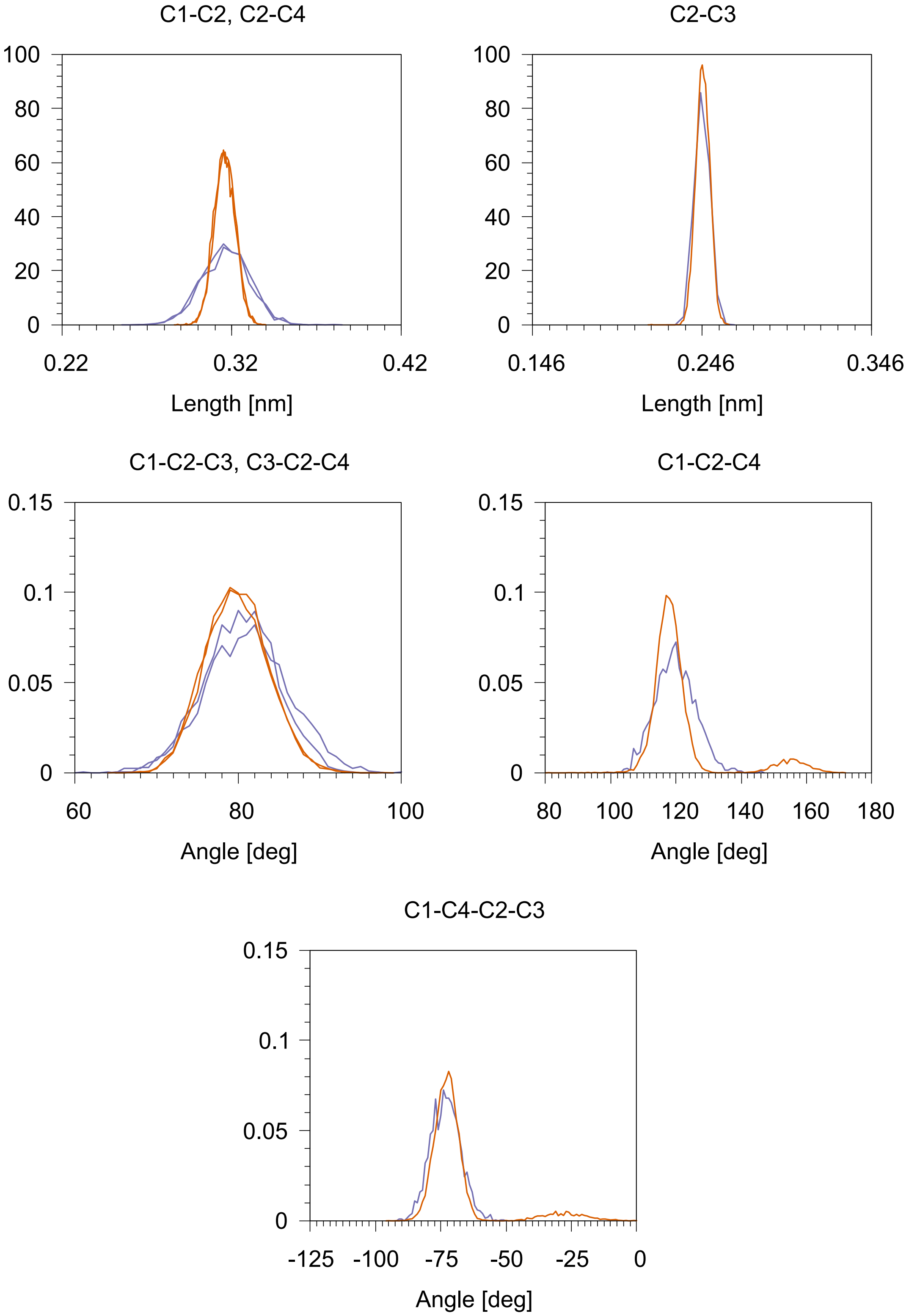

Figure S3. Distributions of the bond length (first row), angle (second row) and dihedral angle (last row) between citrate beads (see Figure S1 for bead numbering). Violet represents the M2 Martini model, in which the citrate molecule has a net charge of –2*e;* in orange the atomistic all-atom model (OPLS).

Citrate dimerization. The PMF windows show that in the dimer configuration one or more ions form bridges between the two citrates. Reducing the overall charge of the citrate when passing from

the model M3 (with charge 3-) to the model M2 (with charge 2-), we managed to significantly reduce the presence of the ion bridges, as shown in figure S4.

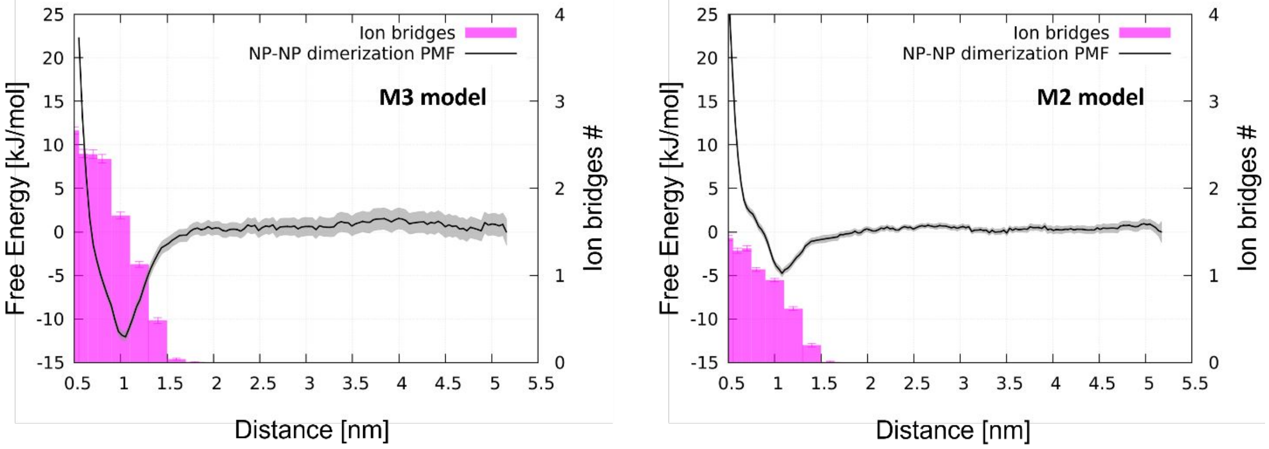

Figure S4. Free energy dimerization profiles for the two tested citrate CG models (with 3 or 2 overall negative charge), with average number of ion bridges per US window. The bridges are defined as ion beads simultaneously in contact (distance < 0.8 nm) with a bead of the two citrate molecules in solution.

	External reference	OPLS	M2
Water-Octanol Partitioning coefficient	-1.3[33],-0.55[34]		-2.0
Citrate-Citrate dimerization distance		0.63±0.01 nm	1.07±0.05 nm
Citrate-Citrate dimerization energy		-5.9±0.1 kJ/mol	-4.8±0.4 kJ/mol
Citrate-POPC adhesion energy		No minimum	-6.6±1.4 kJ/mol
Citrate-Au adhesion energy		-55.7±0.8 kJ/mol	-46.5±0.5 kJ/mol

Table S3. Performance of the coarse grained citrate model vs. the target properties used at development stage. M2 refers to the final model with 2 negative charges.

4.2 Development of the CG Au model

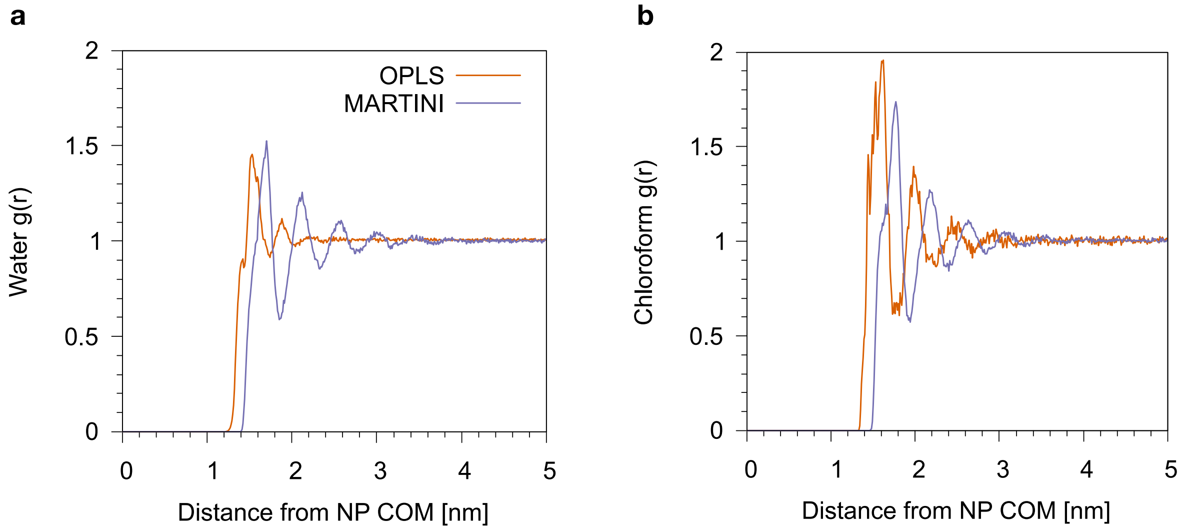

Figure S5. Gold–water (**a**) and gold–chloroform (**b**) RDF, *g(r)*, as a function of the distance from the NP COM. The NP used at both atomistic and Martin level has a diameter of ~ 2.5 nm with a truncated octahedron core structure.

Citrate adsorption on Au surfaces: OPLS vs. C36

In both OPLS and C36 forcefields, citrate spontaneously and stably binds to Au atoms with three possible binding geometries, as shown in Figure S6c. In configuration (1) a water layer is present between the gold surface and the oxygen atoms of the carboxylate groups. In configuration (2) the oxygen atoms of the carboxylate groups are in direct contact with the surface. Finally, in configuration (3) the CH2 groups of the citrate backbone lay on Au atoms. Contrary to the OPLS, the C36 forcefield captures a stable bound state only for the conformation (1) and (3), for which the free energy differences are: $\Delta G^{(1)} = –4 \pm 3$ kJ mol^{-1} and $\Delta G^{(3)} = –37 \pm 3$ kJ mol^{-1} for the polarizable Au model and $\Delta G^{(1)} = –7 \pm 2$ kJ mol^{-1} and $\Delta G^{(3)} = –45 \pm 2$ kJ mol^{-1} for the non-polarizable model. A stable bound state for the conformation (2) is not captured at all for the C36 forcefield. Instead, the C36 forcefield with non-polarizable Au model capture a further conformation, that differs from the conformation (2) for a water layer that is still present between the carboxyl oxygens and Au atoms.

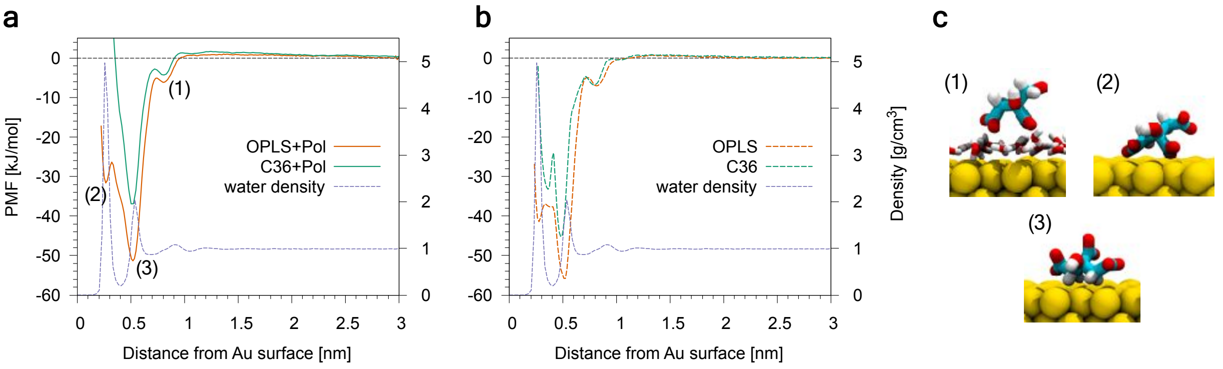

Figure S6. Potential of mean force (PMF) profile of the adsorption of one citrate molecule on a Au (111) surface with the OPLS or CHARMM36 force field in combination with the polarizable gold model (**a**) or with the non-polarizable gold model (**b**) as a function of the distance from the

Au surface. **c.** Binding geometries of one citrate molecule on top of a Au (111) surface at atomistic level.

In particular, Al-Johani and coworkers[35], by combining solid-state NMR, XPS, and TEM experiments with ab-initio DFT calculations in vacuum, have proposed a more detailed picture of the structure and modes of interaction of citrate molecules on top of a Au (111) surface. They conclude that between the various binding geometries, carboxylate-containing ligands bind to the Au surface with the carboxylate oxygens. For citrate, there are three possible configurations: monocarboxylate monodentate, monocarboxylate bridging, and dicarboxylate bridging. In this context, only the OPLS forcefield samples the bound state with the carboxyl oxygens in contact with gold, *i.e.* citrate in the conformation (2), though the free energy profile show that it is a metastable configuration with an escaping free energy barrier of only a few k_BT.
These considerations suggest that atomistic force field could be refined in this respect. Nevertheless, the difference between (3) and (2) can not be captured at CG level.

4.3 Summary of the new model

Final interactions for the new citrate and gold models, to be used in combination with the CG Martini force field[36].

	Qa	Q0	P4	Na	C4	C3	C1	AU	NCIT	QCIT
AU	VIII	V	**σ = 0.401 nm ε = 1.0 kJ/mol**	VII	**σ = 0.47 nm ε = 1.5 kJ/mol**	VII	VII	ref[18]	VI	VIII
NCIT	III	VI	III	II	V	VI	VI	VI	II	I
QCIT	II	V	0	III	VI	VII	IX	VIII	I	I

Table S4. The new interaction matrix of Martini beads used in this work. Interaction levels: O: 5.6 kJ mol^{-1},I: 5.0 kJ mol^{-1}, II: 4.5 kJ mol^{-1}, III: 4.0 kJ mol^{-1}, IV: 3.5 kJ mol^{-1}, V: 3.1 kJ mol^{-1}, VI: 2.7 kJ mol^{-1}, VII: 2.3 kJ mol^{-1}, VIII: 2.0 kJ/mol^{-1} all with σ = 0.47 nm; and, IX: e = 2.0 kJ mol^{-1}, σ = 0.62 nm. We remind that CG water beads are type P4 beads.

5. Model validation

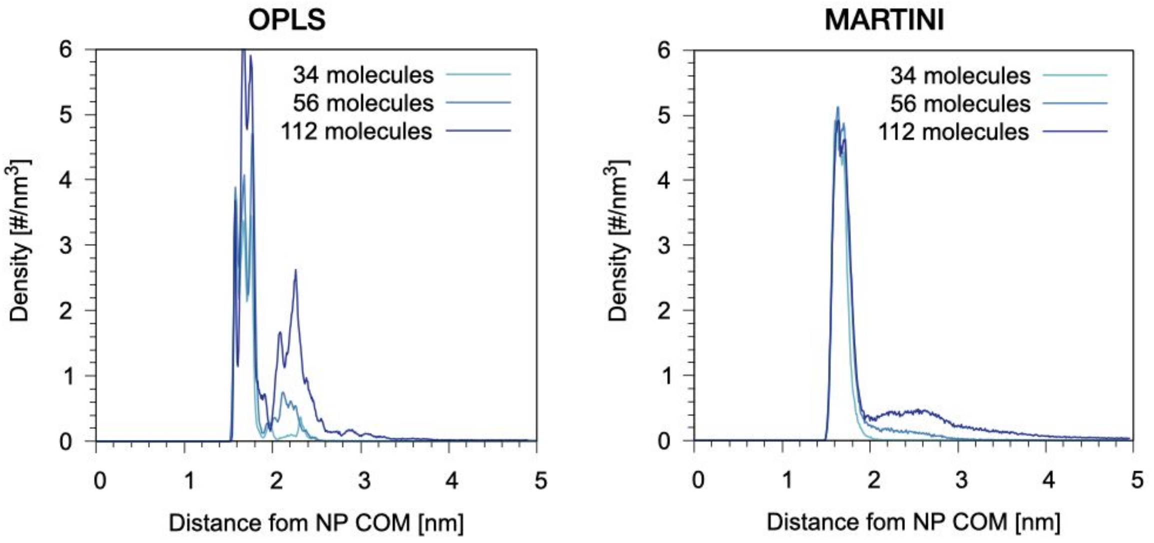

Figure S7. Radial number density of citrate molecules respect to the NP COM for OPLS and Martini for the different citrate concentrations.

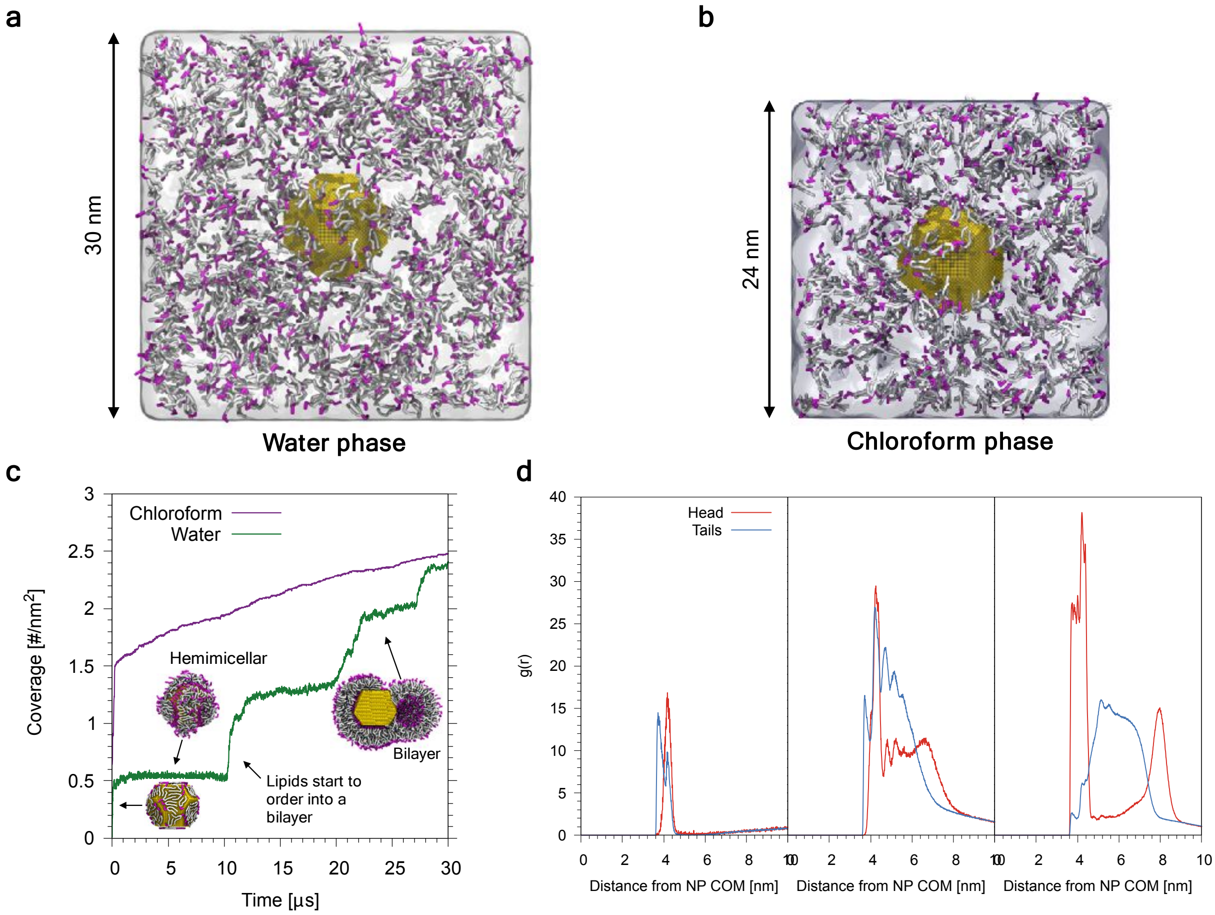

Figure S8. Starting configuration of the self-assembly simulations with a truncated octahedron NP with a core diameter of about 8 nm in water (**a**) or chloroform (**b**). The POPC lipids are randomly placed in the simulation box. **c)** Total number of POPC lipids whose head is within 0.8 nm from any Au atoms normalized by the NP area as a function of the time. **d)** Radial distribution function, *g(r)*, of the lipid heads or tails as a function of the distance from the NP COM, for the three different phases: lipids laying on NP surface, hemimicellar phase and lipid bilayer final phase.

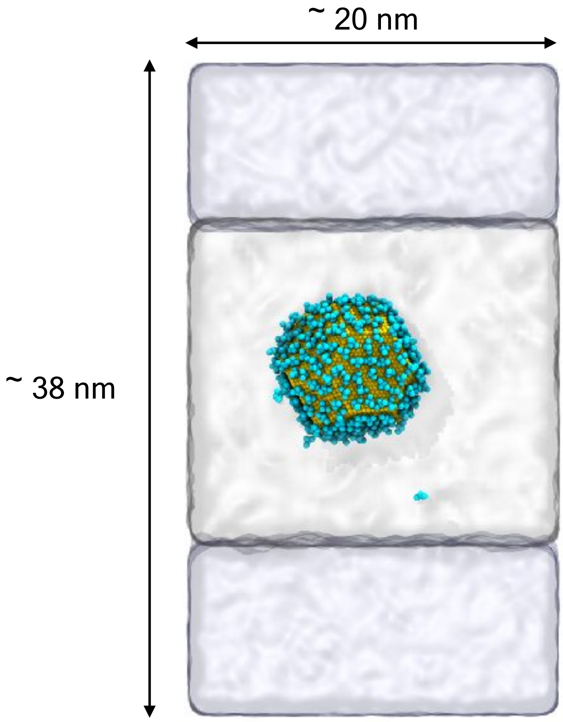

Figure S9. Water/chloroform biphasic system with a citrate-capped Au NP (with a truncated octahedron core structure and diameter of about 8 nm) dissolved in the water phase. After 10 ms the Au NP still remain in the water phase. Chloroform and water chambers shown as light-blue and light-gray shaded area. Citrate molecules in cyan and Au atoms in yellow.

6. Test application

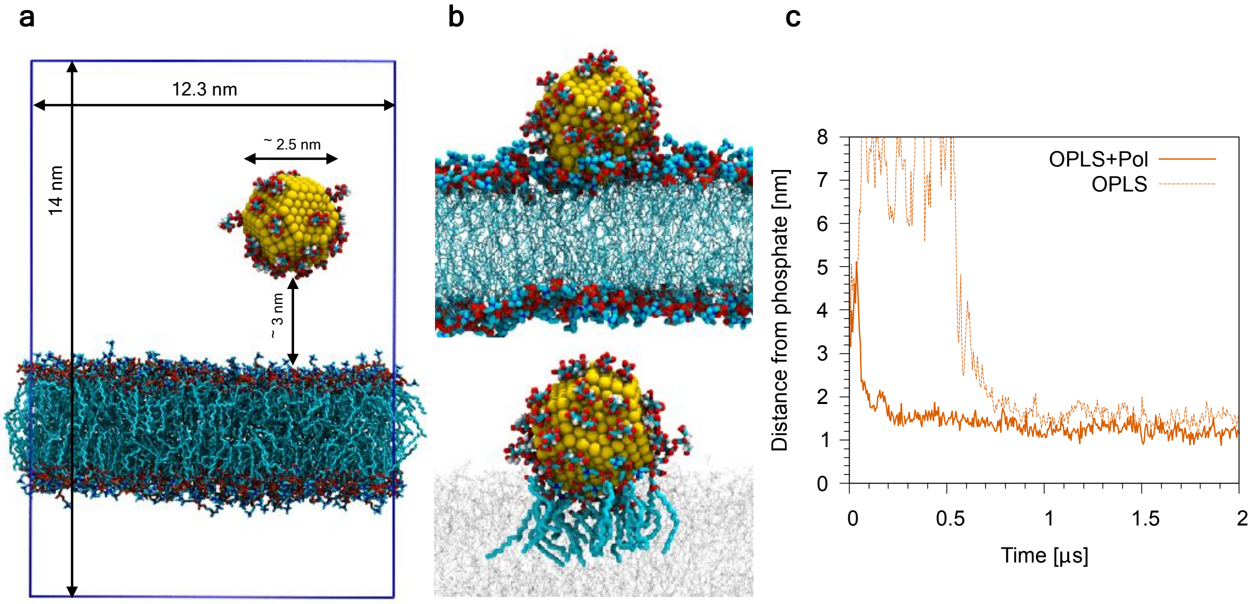

Figure S10. Stable adsorption of a citrate-capped Au NP onto a zwitterionic POPC bilayer at atomistic level. The Au NP has a truncated octahedron core structure with a diameter of about 2.5 nm. Initial (**a**) and final (**b**) configuration, and the plot of the z-distance between the NP COM and the bilayer phosphate group as a function of the simulation time (**c**). In the final configuration snapshot, the lipid whose nitrogen or phosphorus atoms are within 0.6 nm from any Au atoms are shown as thicker and colored sticks while the other lipids as gray thin sticks.

We first studied the interaction between citrate-capped Au NPs (CNPs) and model zwitterionic lipid bilayer (POPC) at the atomistic level. We only used the truncated octahedron Au NP with a core diameter of ~ 2.5 nm. The citrate coverage in the initial configuration is 1.67 molecules nm^{-2} (a total of 34 citrate molecules) and only the citrate molecules in direct contact with Au are kept with their counterions. In the initial configuration (see Figure S10), the CNP is placed in the water phase above a preformed zwitterionic lipid bilayer. After about 100 ns the CNP from the water phase spontaneously and stably adheres to the surface of the lipid bilayer, on the side of the NP which is not covered by citrate molecules. Here, the lipid head groups are in direct contact with Au atoms, as shown in the final snapshot of the simulation in Figure S10. Looking at the distance between the COM of the bilayer and the COM of the NP, along the membrane normal, the CNP is stably bound to the bilayer for more than 1.5 ms, over a 2 ms long trajectory. We have also tested the non-polarizable Au model and the behavior is almost similar, but the CNP seems to be slightly less deep into the bilayer, respect to the simulation with the polarizable Au model (see Figure S10). The configuration in which the CNP adhere on top of the lipid bilayer seems to be a metastable state. Unfortunately, the accessible time scale at the atomistic level does not allow to overcome the free energy barriers that separates the configuration in which the CNP is completely wrapped by the bilayer. Moreover, the simulated time is not enough to observe the exchange reaction to occur. The limited accessible length scale with atomistic simulations makes it impossible to study the CNP–membrane interaction with NP size comparable to that used in experiments (on the order of tens of nm). This is more indication of the necessity of the use and development of the CG Martini model for the citrate-Au NP system, in order to overcome these limitations.

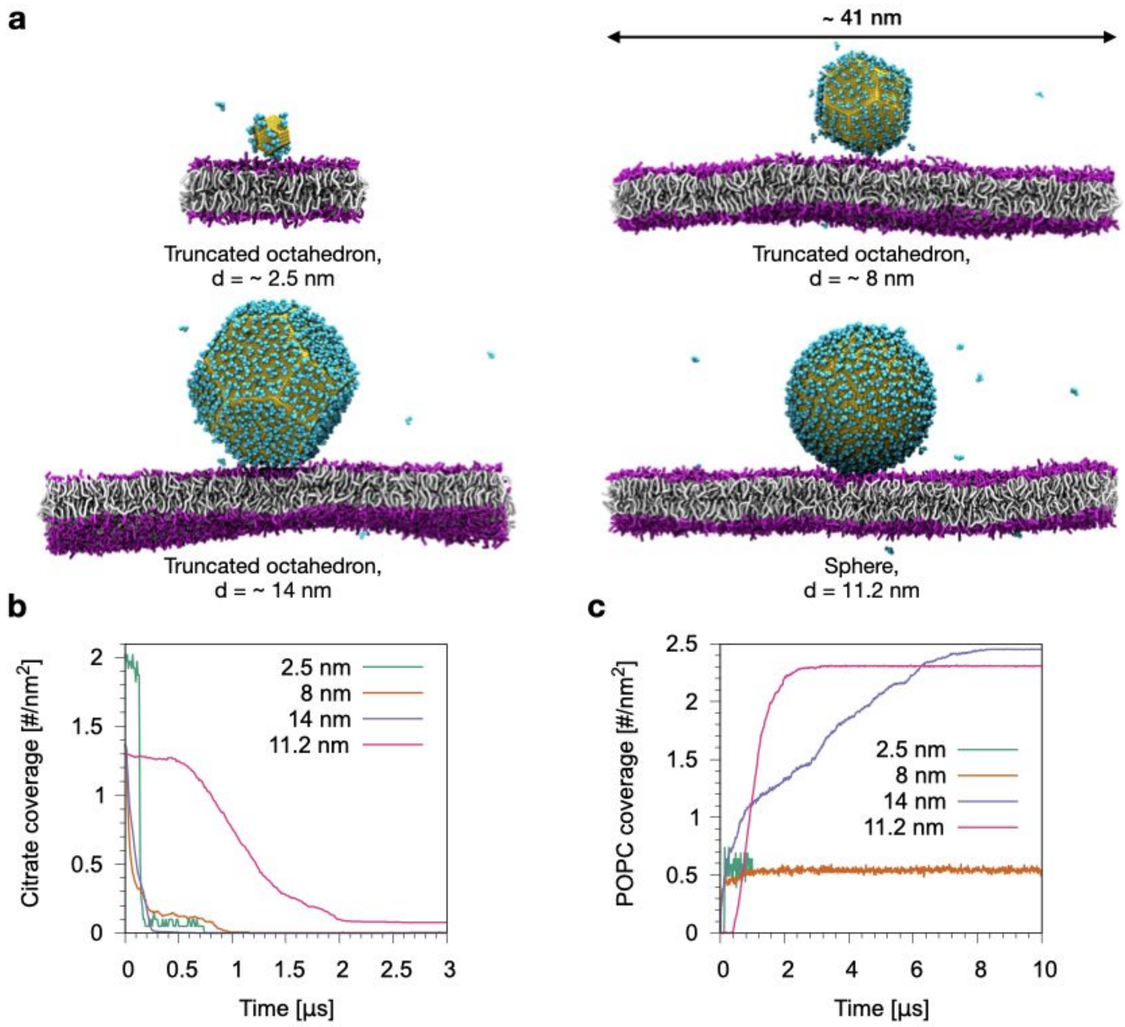

Figure S11. a Adsorbed configuration for two different NP types: a citrate layer remains in between the Au surface and the lipid headgroups. **b** Plot of the citrate coverage (number of adsorbed citrates over the area of the given NP) as a function of the time. **c** Plot of lipid coverage (number of POPC lipids whose head is within 0.8 nm from Au atoms over the area of the given NP) as a function of the time.

Experimental Methods

AuNP Characterization. The size and concentration of AuNPs were estimated from UV-Vis spectroscopy, with a JASCO UV-Vis spectrophotometer.

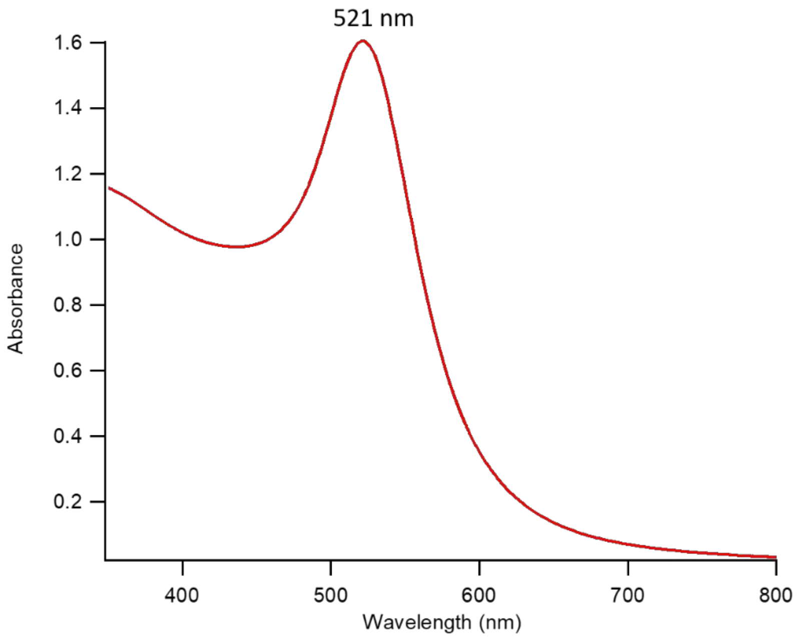

Figure S12 UV-Vis absorption spectrum of citrate-stabilized gold nanoparticles in water after 1:3 dilution. The plasmon absorption peak is highlighted and falls at 521 nm.

The size of AuNPs was evaluated by the following equation[37]:

$$d = \exp\left(B_1 \frac{A_{spr}}{A_{450}} - B_2\right)$$

with d diameter of gold nanoparticles, A_{spr} absorbance at the surface plasma resonance peak, A_{450} absorbance at the wavelength of 450 nm and B_1 and B_2 dimensionless parameters, taken as 3 and 2.2, respectively. The diameter value obtained is 15.2 nm. The concentration of AuNPs was then determined, using the Lambert-Beer law (i.e., $A(\lambda) = \varepsilon(\lambda)lc$, with $A(\lambda)$ absorbance at the maximum of the resonance peak, l optical length, c concentration of AuNPs and ε (λ) the extinction value at the LSPR maximum, i.e. λ = 521 nm). The extinction coefficient $\varepsilon(\lambda)$ of gold nanoparticles was determined by the following equation[38]:

$$\ln(\varepsilon) = k\ln(d) + a$$

with d diameter of nanoparticles and k and a dimensionless parameters (k=3.32111 and a=10.80505). To the purpose, the size obtained by UV-Vis spectroscopy analysis was selected, leading to $4.1 \cdot 10^8$ $M^{-1}cm^{-1}$. The final concentrations of AuNPs is $7.7 \cdot 10^{-9}$ M.

Liposomes characterization. Liposomes were characterized through Dynamic Light Scattering (Malvern Panalytical Zetasizer Nano ZS90 instrument). Measurements were performed at a fixed scattering angle of 90°, with a 4 mW laser of 633 nm wavelength as light source; the lag times of the correlator start from 25 ns as shortest and go up to 8000 s, using a maximum number of 4000 channels. After checking monomodality with a CONTIN fit, the Autocorrelation Functions were analyzed through the cumulant fitting limited to the second order, allowing an estimate of the hydrodynamic diameter and the polydispersity of POPC liposomes, which were found equal to: 99 ± 3 nm (hydrodynamic diameter) with a 0.12 PDI.

ATR-FTIR spectroscopy. ATR-FTIR analysis was performed on dry films using a Thermo Nicolet Nexus 870 with an MCT detector (Mercury Cadmium Tellurium). 10 µL of liquid sample were deposited on the germanium crystal and dried under nitrogen flux. To the purpose, freshly

synthetized AuNPs in water were selected, without further modification. AuNPs in CHCl3 were directly collected from the organic phase of the water/CHCl3 biphasic assay (POPC concentration of 1 mg/ml), after centrifugation. A reference spectrum for POPC was obtained evaporating a POPC solution in CHCl3 at 1 mg/ml concentration. Spectra were acquired after full evaporation of the solvent, in the range 4000-649 cm-1, with a spectral resolution of 4 cm-1 and 128 scans for each spectrum.

References

1. Jorgensen, W. L., Maxwell, D. S. & Tirado-Rives, J. Development and testing of the OPLS all-atom force field on conformational energetics and properties of organic liquids. *J. Am. Chem. Soc.* **118**, 11225–11236 (1996).
2. Jorgensen, W. L., Madura, J. D. & Swenson, C. J. Optimized intermolecular potential functions for liquid hydrocarbons. *J. Am. Chem. Soc.* **106**, 6638–6646 (1984).
3. Briggs, J. M., Matsui, T. & Jorgensen, W. L. Monte Carlo simulations of liquid alkyl ethers with the OPLS potential functions. *J. Comput. Chem.* **11**, 958–971 (1990).
4. Kaminski, G. A., Friesner, R. A., Tirado-Rives, J. & Jorgensen, W. L. Evaluation and Reparametrization of the OPLS-AA Force Field for Proteins via Comparison with Accurate Quantum Chemical Calculations on Peptides †. *J. Phys. Chem. B* **105**, 6474–6487 (2001).
5. Wright, L. B., Rodger, P. M. & Walsh, T. R. Aqueous citrate: a first-principles and force-field molecular dynamics study. *RSC Adv.* **3**, 16399 (2013).
6. Vanommeslaeghe, K. *et al.* CHARMM general force field: A force field for drug-like molecules compatible with the CHARMM all-atom additive biological force fields. *J. Comput. Chem.* NA-NA (2009). doi:10.1002/jcc.21367
7. Yu, W., He, X., Vanommeslaeghe, K. & MacKerell, A. D. Extension of the CHARMM general force field to sulfonyl-containing compounds and its utility in biomolecular simulations. *J. Comput. Chem.* **33**, 2451–2468 (2012).
8. Shafeeuulla Khan, M. A., Sen, A. & Ganguly, B. Probing the influence of pH dependent citric acid towards the morphology of rock salt: a computational study. *CrystEngComm* **11**, 2660 (2009).
9. MacKerell, A. D. *et al.* All-Atom Empirical Potential for Molecular Modeling and Dynamics Studies of Proteins †. *J. Phys. Chem. B* **102**, 3586–3616 (1998).
10. Park, J. W. & Shumaker-Parry, J. S. (sup)Structural study of citrate layers on gold nanoparticles: Role of intermolecular interactions in stabilizing nanoparticles. *J. Am. Chem. Soc.* **136**, 1907–1921 (2014).
11. Wright, L. B., Rodger, P. M., Corni, S. & Walsh, T. R. GolP-CHARMM: First-Principles Based Force Fields for the Interaction of Proteins with Au(111) and Au(100). *J. Chem. Theory Comput.* **9**, 1616–1630 (2013).
12. Perfilieva, O. A., Pyshnyi, D. V. & Lomzov, A. A. Molecular Dynamics Simulation of Polarizable Gold Nanoparticles Interacting with Sodium Citrate. *J. Chem. Theory Comput.* **15**, 1278–1292 (2019).
13. Dodda, L. S., Vilseck, J. Z., Tirado-Rives, J. & Jorgensen, W. L. 1.14*CM1A-LBCC: Localized Bond-Charge Corrected CM1A Charges for Condensed-Phase Simulations. *J. Phys. Chem. B* **121**, 3864–3870 (2017).
14. Dodda, L. S., De Vaca, I. C., Tirado-Rives, J. & Jorgensen, W. L. LigParGen web server: An automatic OPLS-AA parameter generator for organic ligands. *Nucleic Acids Res.* **45**, W331–W336 (2017).
15. Jorgensen, W. L. & Tirado-Rives, J. Potential energy functions for atomic-level simulations of water and organic and biomolecular systems. *Proc. Natl. Acad. Sci.* **102**, 6665–6670 (2005).
16. Berger, O., Edholm, O. & Jahnig, F. Molecular Dynamics Simulations of a Fluid Bilayer of Dipalmitoylphosphatidylcholine at Full Hydration, Constant Pressure, and Constant

Temperature. *Biophys. J.* **72**, 2002–2013 (1997).
17. Tieleman, D. P. *et al.* Membrane protein simulations with a united-atom lipid and all-atom protein model: lipid-protein interactions, side chain transfer free energies and model proteins. *J Phys Condens Matter* **18**, S1221-34 (2006).
18. Heinz, H., Vaia, R. A., Farmer, B. L. & Naik, R. R. Accurate simulation of surfaces and interfaces of FCC metals using 12–6 and 9–6 Lennard-Jones potentials. *J. Phys. Chem.* **112**, 17281 (2008).
19. Geada, I. L., Ramezani-Dakhel, H., Jamil, T., Sulpizi, M. & Heinz, H. Insight into induced charges at metal surfaces and biointerfaces using a polarizable Lennard–Jones potential. *Nat. Commun.* **9**, 716 (2018).
20. BioMembNP repository.
21. Jo, S., Kim, T., Iyer, V. G. & Im, W. CHARMM-GUI: A web-based graphical user interface for CHARMM. *J. Comput. Chem.* **29**, 1859–1865 (2008).
22. Michaud-Agrawal, N., Denning, E. J., Woolf, T. B. & Beckstein, O. MDAnalysis: A toolkit for the analysis of molecular dynamics simulations. *J. Comput. Chem.* **32**, 2319–2327 (2011).
23. Gowers, R. *et al.* MDAnalysis: A Python Package for the Rapid Analysis of Molecular Dynamics Simulations. in *Proceedings of the 15th Python in Science Conference* (eds. Benthall, S. & Rostrup, S.) 98–105 (2016). doi:10.25080/Majora-629e541a-00e
24. Wassenaar, T. A., Ingoífsson, H. I., Bö Ckmann, R. A., Peter, D. & Marrink, S. J. Computational Lipidomics with insane: A Versatile Tool for Generating Custom Membranes for Molecular Simulations. (2015). doi:10.1021/acs.jctc.5b00209
25. Leach, A. R. Molecular Modeling: Principles and Applications 2nd Ed. *Journal of Chemical Information and Computer Sciences* (2002).
26. Bennett, C. H. Efficient estimation of free energy differences from Monte Carlo data. *J. Comput. Phys.* **22**, 245–268 (1976).
27. Kästner, J. Umbrella sampling. *Wiley Interdiscip. Rev. Comput. Mol. Sci.* **1**, 932–942 (2011).
28. Hub, J. S., De Groot, B. L. & Van Der Spoel, D. g_whamsA Free Weighted Histogram Analysis Implementation Including Robust Error and Autocorrelation Estimates. 3713–3720 (2010). doi:10.1021/ct100494z
29. Laio, A. & Parrinello, M. Escaping free-energy minima. *Proc. Natl. Acad. Sci. U. S. A.* **99**, 12562–6 (2002).
30. Barducci, A., Bussi, G. & Parrinello, M. Well-Tempered Metadynamics: A Smoothly Converging and Tunable Free-Energy Method. *Phys. Rev. Lett.* **100**, 020603 (2008).
31. Tribello, G. A., Bonomi, M., Branduardi, D., Camilloni, C. & Bussi, G. PLUMED 2: New feathers for an old bird. *Comput. Phys. Commun.* **185**, 604–613 (2014).
32. Abraham, M. J. *et al.* Gromacs: High performance molecular simulations through multi-level parallelism from laptops to supercomputers. *SoftwareX* **1–2**, 19–25 (2015).
33. Tetko, I. V *et al.* Virtual computational chemistry laboratory--design and description. *J. Comput. Aided. Mol. Des.* **19**, 453–463 (2005).
34. Chemaxon.
35. Al-Johani, H. *et al.* The structure and binding mode of citrate in the stabilization of gold nanoparticles. *Nat. Chem.* **9**, 890–895 (2017).
36. Marrink, S. J., Risselada, H. J., Yefimov, S., Tieleman, D. P. & de Vries†, A. H. The MARTINI Force Field: Coarse Grained Model for Biomolecular Simulations. *J. Phys. Chem. B* **111**, 7812–7824 (2007).
37. Haiss, W., Thanh, N. T. K., Aveyard, J. & Fernig, D. G. Determination of size and concentration of gold nanoparticles from UV-Vis spectra. *Anal. Chem.* **79**, 4215–4221 (2007).
38. Liu, X., Atwater, M., Wang, J. & Huo, Q. Extinction coefficient of gold nanoparticles with different sizes and different capping ligands. *Colloids Surfaces B Biointerfaces* **58**, 3–7

(2007).
